# Leaf microbiome dysbiosis triggered by T2SS-dependent enzyme secretion from opportunistic *Xanthomonas* pathogens

**DOI:** 10.1038/s41564-023-01555-z

**Published:** 2024-01-03

**Authors:** Sebastian Pfeilmeier, Anja Werz, Marine Ote, Miriam Bortfeld-Miller, Pascal Kirner, Andreas Keppler, Lucas Hemmerle, Christoph G. Gäbelein, Gabriella C. Petti, Sarah Wolf, Christine M. Pestalozzi, Julia A. Vorholt

**Affiliations:** 1 Institute of Microbiology, ETH Zurich, Zurich, Switzerland; 2 Molecular Plant Pathology, Swammerdam Institute of Life Sciences, University of Amsterdam, Amsterdam, the Netherlands

**Keywords:** Microbe, Microbiome

## Abstract

In healthy plants, the innate immune system contributes to maintenance of microbiota homoeostasis, while disease can be associated with microbiome perturbation or dysbiosis, and enrichment of opportunistic plant pathogens like *Xanthomonas*. It is currently unclear whether the microbiota change occurs independently of the opportunistic pathogens or is caused by the latter. Here we tested if protein export through the type-2 secretion system (T2SS) by *Xanthomonas* causes microbiome dysbiosis in *Arabidopsis thaliana* in immunocompromised plants. We found that *Xanthomonas* strains secrete a cocktail of plant cell wall-degrading enzymes that promote *Xanthomonas* growth during infection. Disease severity and leaf tissue degradation were increased in *A. thaliana* mutants lacking the NADPH oxidase RBOHD. Experiments with gnotobiotic plants, synthetic bacterial communities and wild-type or T2SS-mutant *Xanthomonas* revealed that virulence and leaf microbiome composition are controlled by the T2SS. Overall, a compromised immune system in plants can enrich opportunistic pathogens, which damage leaf tissues and ultimately cause microbiome dysbiosis by facilitating growth of specific commensal bacteria.

## Abstract (web summary)

Opportunistic plant pathogen *Xanthomonas* secretes leaf-degrading enzymes through the type-2 secretion systems that cause disease and thereby trigger changes in leaf microbiome composition.

## Main

Host-associated microbial communities, collectively referred to as microbiota, promote development, growth and adaptation to abiotic and biotic stress in healthy host organisms. Bacteria are abundant members in the microbiota and assemble into taxonomically structured communities in animals and plants^1–3^.

Under certain circumstances, the relationship between the host and its microbiota can become unbalanced, resulting in an alternative state of the microbial community termed dysbiosis, which is commonly associated with disease and with an alteration in the composition or function of the microbiome^4,5^. The host immune system plays a central role in maintaining and controlling microbiota homoeostasis to prevent dysbiosis^4,6^. In addition, opportunistic pathogens are particularly relevant in dysbiosis as they are normally harmless for the host but are equipped with potential virulence functions and, under conducive conditions, eventually cause context-dependent diseases. In mammals, opportunistic pathogens present in the gut or on the skin have been associated with disease in hosts that have a compromised immune system and have a reduced microbiota diversity^4,7,8^. Therefore, dysbiosis has underlying contributions both from individual species with pathogenic potential and from the microbiota.

Dysbiosis can also occur in plant leaf microbiota^9,10^. A reverse genetic screen in *Arabidopsis thaliana* mutants with defects in the immune system revealed that *rbohD* knockout plants, among others, harbour an altered phyllosphere microbiota and develop disease^9^. In this case, two *Xanthomonas* strains were identified as opportunistic pathogens in *rbohD* plants and as the driver of plant disease after inoculation with a bacterial synthetic community (SynCom) of more than 200 strains that contained these opportunistic pathogens^9^. The two *Xanthomonas* strains, Leaf131 and Leaf148, are part of the representative *At*-LSPHERE strain collection^1^ and were recently placed into distinct phylogenetic clades, that is *Xanthomonas hortorum* and *Xanthomonas dyei*, respectively^11^. Both strains lack a type-3 secretion system, a typical virulence factor of bona fide pathogens, which might render them non-virulent on *A. thaliana* Col-0 wild type. Opportunistic *Xanthomonas* in plants have been reported previously to cause soft rot in wounded plant tissue due to their pectolytic activity^12,13^.

In plants, the NADPH oxidase RBOHD produces apoplastic reactive oxygen species (ROS) and is involved in several pathways related to growth, development and stress response^14–16^. Moreover, RBOHD is an important component of the plant immune system^17^. Plants recognize microorganisms due to microbe- or danger-associated molecular patterns or microbial effector proteins that lead to activation of RBOHD, which is a convergence point of pattern-triggered immunity and effector-triggered immunity signalling pathways^18^. RBOHD-produced ROS also function in cell wall polymer crosslinking during pathogen-induced lignification^19,20^. Apart from plants, other multi-cellular organisms possess NADPH oxidases, including fungi, where they serve both defence and differentiation signalling^21^, and in mammals^14,22^, they are involved in gut epithelial immune responses and prevent intestinal dysbiosis^23,24^.

In this study, we dissect the contribution of opportunistic *Xanthomonas* strains, their context-dependent virulence and host genotype to the bacterial community composition in the phyllosphere of *A. thaliana* mutants defective in RBOHD. We used a SynCom approach, which has emerged as a decisive tool to study the processes and interactions shaping the microbiota and affecting the host^9,25–29^, and both targeted and random bacterial mutagenesis. Our results link plant immunity to dysbiosis by establishing a causal relationship between a plant protein (RBOHD) and a bacterial trait (enzyme secretion via T2SS) within a rather complex microbiome.

## Dysbiosis caused by opportunistic *Xanthomonas* in *rbohD* plants

*A. thaliana* plants with defective RBOHD, but not wild-type plants, show impaired growth and disease when inoculated with a synthetic community and exhibited a dysbiotic microbiota. The *rbohD* phenotype can be remediated by removing the *Xanthomonas* Leaf131 strain from a 137-member microbiota community^9^. To determine whether the opportunistic pathogen not only drives plant disease but also alters the microbiota composition in *rbohD* plants, we inoculated microbiota-free *A. thaliana* seedlings with a SynCom of 137 strains that did or did not include *Xanthomonas* Leaf131 and analysed the community composition on Col-0 wild type, *rbohD* knockout and the complementation line *rbohD/RBOHD* by 16S ribosomal RNA (rRNA) amplicon sequencing.

**Fig. 1:**
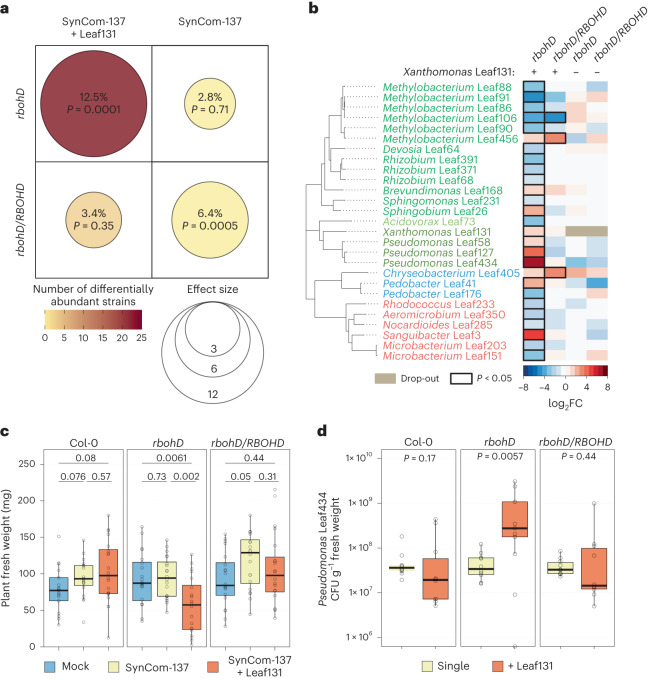
Microbiota shift and plant disease driven by *Xanthomonas* Leaf131 in *rbohD* knockout plants. **a**, Composition of synthetic bacterial communities SynCom-137 + *Xanthomonas* Leaf131 or SynCom-137 in *rbohD* or *rbohD*/*RBOHD* plants was compared with Col-0 wild-type plants. Effect size represents percentage of total variance explained by genotype (shown by dot size and absolute value) and statistical significance is expressed with *P* values determined by PERMANOVA (Benjamini–Hochberg adjusted, *n* = 16). Number of differentially abundant strains (as shown in **b**) is shown by dot colour. **b**, Heatmap shows subset of strains in SynCom-137 with significant log_2_ fold changes (log_2_FC, *P* < 0.05) in *rbohD* or *rbohD*/*RBOHD* compared with Col-0 wild-type plants in the presence (+) or absence (−) of *Xanthomonas* Leaf131. Black rectangles show significant changes, *P* < 0.05 (*n* = 16, two-sided Wald test, Benjamini–Hochberg adjusted). Complete heatmap of all strains in SynCom-137 is shown in Extended Data Fig. 1c. **c**, Fresh weight of aboveground plant tissue of Col-0, *rbohD* and *rbohD*/*RBOHD* mock inoculated, with SynCom-137 or SynCom-137 + *Xanthomonas* Leaf131. Box plots show the median with upper and lower quartiles and whiskers present 1.5× interquartile range (*n* = 16, two-sided Mann–Whitney *U* test, *P* values indicated above box plots). Corresponding plant phenotypes are shown in Extended Data Fig. 1d. **d**, CFU counts of *Pseudomonas* Leaf434 per gram plant fresh weight after inoculation of germ-free Col-0, *rbohD* and *rbohD/RBOHD* plants with *Pseudomonas* Leaf434 as single inoculation or in binary inoculation with *Xanthomonas* Leaf131 Tn7::Gm-lux. Box plots show the median with upper and lower quartiles and whiskers present 1.5× interquartile range (*n* = 12, two-sided Mann–Whitney *U* test, *P* values indicated above box plots). Source data

**Extended Data Fig. 1:**
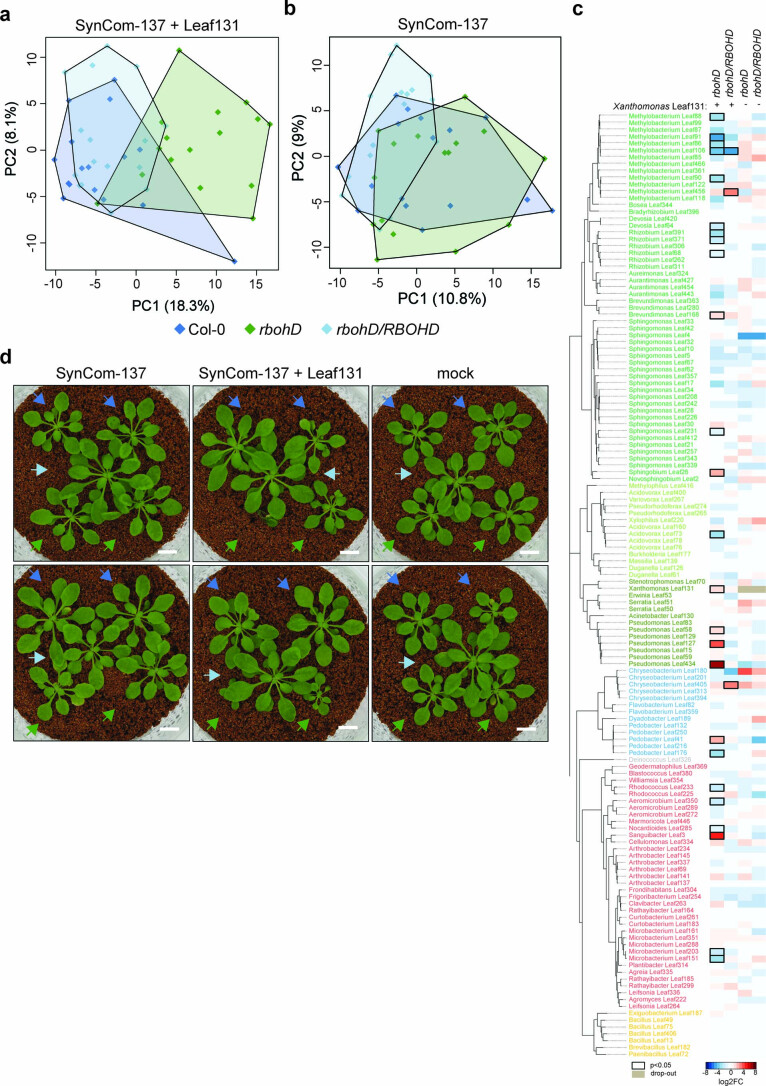
Microbiota composition of SynCom-137 and plant phenotype in presence and absence of *Xanthomonas* Leaf131. **a)** Principal component analysis of SynCom-137+*Xanthomonas* Leaf131 and of **b)** SynCom-137 in Col-0 (blue), *rbohD* (green) and *rbohD/RBOHD* (light blue). Axes show principal components PC1 and PC2 with their explained variance (%). Statistical analysis (PERMANOVA) analysis is represented by effect size shown in Fig. 1A**c)** Heatmap shows log_2_ fold changes (log2FC) of strains in SynCom-137 in *rbohD* or *rbohD*/*RBOHD* compared to Col-0 wild-type plants in the presence (+) or absence (-) of *Xanthomonas* Leaf131. Black rectangles show significant changes, p-value < 0.05 (n = 16, two-sided Wald test, Benjamini–Hochberg adjusted). Subset of same data is shown in Fig. 1b. **d)** Plant phenotype of Col-0 (blue arrow), *rbohD* (green arrow) and *rbohD/RBOHD* (light blue arrow) mock inoculated or with SynCom-137 or SynCom-137+*Xanthomonas* Leaf131. Two representative replica growth boxes are shown for each treatment. Source data

As an indicator for monitoring overall community changes, we used effect size to quantify how much of the total variance in the microbiota is explained by the plant genotype. As expected, we observed that the microbiota composition in *rbohD* plants when compared with Col-0 was significantly altered when *Xanthomonas* Leaf131 was included in the microbiota, that is, SynCom-137+Leaf131 with an effect size of 12.5% (*P* = 0.0001). In contrast, the community composition did not significantly change when Leaf131 was omitted from the SynCom-137 (effect size 2.8%, *P* = 0.71) (Fig. 1a). Consistent with this, the difference in community composition of SynCom-137 in *rbohD* plants was observed when *Xanthomonas* Leaf131 was included, but not in the absence of the opportunistic pathogen, as indicated by a principal component analysis (PCA) (Extended Data Fig. 1a). Analysis of the effect of addition of *Xanthomonas* Leaf131 to the SynCom on the overall community composition for each genotype confirmed the *rbohD*-specific impact (Fig. 1 and Source Data). By analysing the changes in relative abundance of each strain in the SynCom-137, we found that specific strains were enriched in *rbohD* compared with Col-0, resulting in the characteristic microbiota shift in diseased *rbohD* plants as observed previously^9^. In addition to *Xanthomonas* Leaf131, we found that the Gammaproteobacteria *Pseudomonas* Leaf58, Leaf127 and Leaf434, the Alphaproteobacteria *Sphingobium* Leaf26 and *Brevundimonas* Leaf168, the Bacteroides *Pedobacter* Leaf41, as well as the Actinobacterium *Sanguibacter* Leaf3 were enriched in their relative abundance (Fig. 1b and Extended Data Fig. 1c). None of the changes in the relative abundance of these strains could be observed in *rbohD* plants in the absence of *Xanthomonas* Leaf131, which is also a Gammaproteobacterium.

As the SynCom-137 did not show significant differences in community composition in *rbohD* compared with the control Col-0 without *Xanthomonas* Leaf131 (Fig. 1a,b and Extended Data Fig. 1a,b), we conclude that *rbohD* does not affect the microbiota per se, but rather indirectly via *Xanthomonas* Leaf131. Consistently, only *rbohD* knockout plants showed disease symptoms and a reduced average plant fresh weight after inoculation with SynCom137+Leaf131, but not Col-0 or *rbohD/RBOHD* (Fig. 1c and Extended Data Fig. 1d).

To exemplarily validate that certain members of the microbiota benefit from the presence of *Xanthomonas* Leaf131 on *rbohD* plants but not on Col-0 wild-type plants, we selected a commensal strain, *Pseudomonas* Leaf434, that was enriched on *rbohD* plants on the basis of our data from the SynCom experiment (Fig. 1b), and assessed its absolute abundance in a binary inoculation experiment together with *Xanthomonas* Leaf131. Substantiating the results of the SynCom experiment, *Pseudomonas* Leaf434 showed higher plant colonization levels only in *rbohD* plants when inoculated together with *Xanthomonas* Leaf131 compared with single inoculation or in control Col-0 and *rbohD/RBOHD* plants (Fig. 1d).

Overall, our data show that the presence of the opportunistic pathogen *Xanthomonas* Leaf131 leads to dysbiosis and an enrichment, possibly through the promotion of growth, of specific microbiota members in *rbohD* plants.

## Plant tissue degradation by opportunistic *Xanthomonas*

**Fig. 2:**
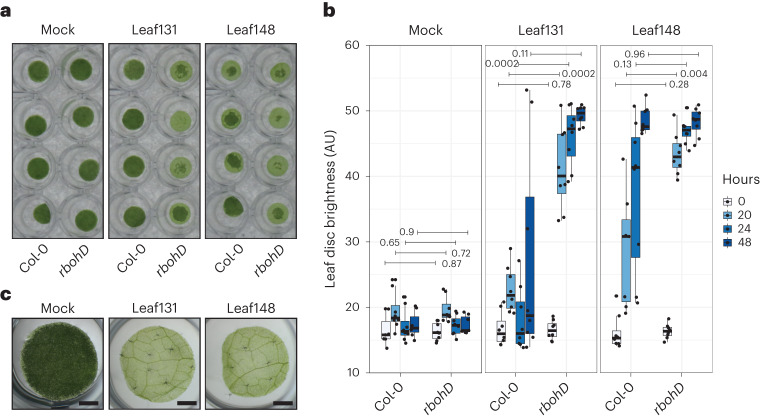
*Xanthomonas* Leaf131 and Leaf148 degrade plant tissue. **a**, Leaf discs of Col-0 and *rbohD* plants (6 weeks old) were mock inoculated (10 mM MgCl_2_) or inoculated with *Xanthomonas* Leaf131 or Leaf148 (OD of 0.02) and incubated for 20 h. **b**, Time-course measurement and quantification of leaf disc brightness (arbitrary unit, AU) from experiment described in **a**. Statistical differences between Col-0 and *rbohD* at varying timepoints are indicated above box plots, with *P* value on the right or left of horizontal line indicating comparison (two-sided Mann–Whitney *U* test, *n* = 8). Box plots show the median with upper and lower quartiles and whiskers present 1.5× interquartile range. **c**, Leaf disc of 5-week-old *rbohD* plants mock (10 mM MgCl_2_) inoculated or with *Xanthomonas* Leaf131 or Leaf148 (OD of 0.02) and incubated for 48 h. Scale bar, 1 mm. Source data

When examining possible virulence mechanisms, we found that *Xanthomonas* Leaf131 and also Leaf148, previously identified as opportunistic pathogens^9^, degrade leaf tissue. We therefore set up a quantitative *A. thaliana* assay to assess tissue degradation using leaf discs (Fig. 2). Both *Xanthomonas* strains degraded the tissue, which we quantified using pixel brightness. We observed that tissue degradation was markedly more severe in leaf discs of *rbohD* plants compared with Col-0 plants (Fig. 2a,b), corroborating the stronger virulence phenotype of these *Xanthomonas* strains in *rbohD* plants^9^.

**Extended Data Fig. 2:**
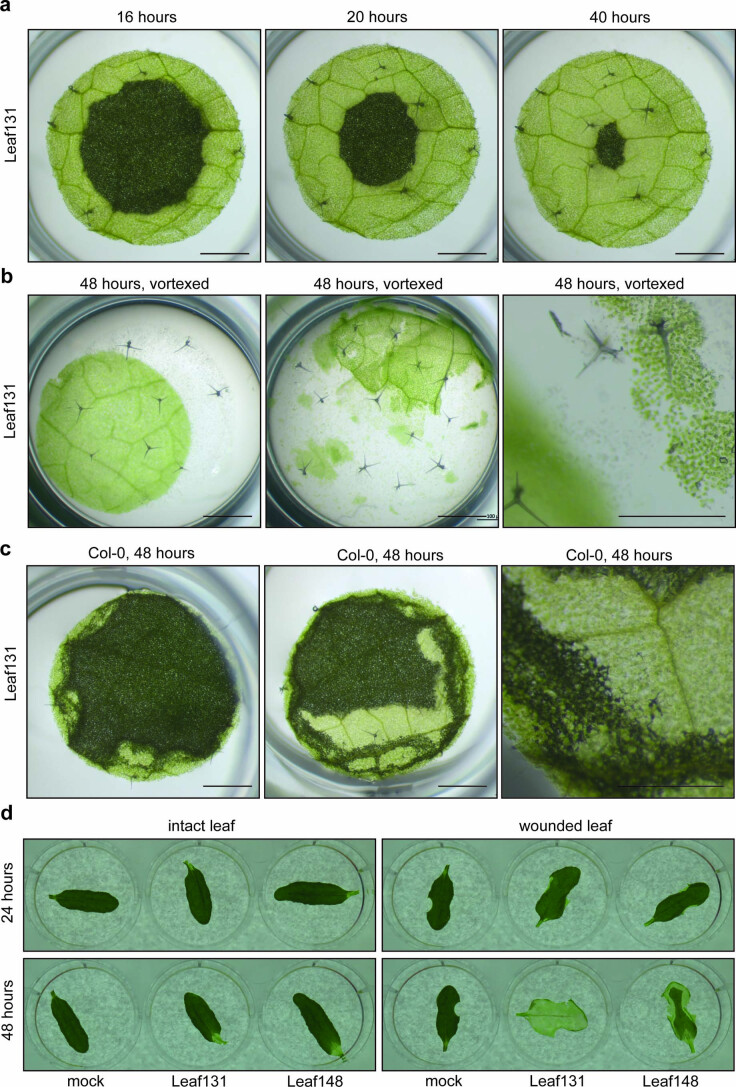
*Xanthomonas* disrupts leaf tissue cohesion and requires plant wound. **a)** Time-course of leaf discs from five-week-old *rbohD* plants inoculated with *Xanthomonas* Leaf131 (OD=0.02). Scale bar represents 1 mm. **b)** Leaf discs of *rbohD* plants in 96-well plate after 48 hours incubation with *Xanthomonas* Leaf131 were vortexed for two seconds. Scale bar represents 1 mm in left and middle panel, and 0.5 mm in right panel. **c)** Leaf discs of five-week-old Col-0 plants after 48 hours incubation with *Xanthomonas* Leaf131. Scale bar represents 1 mm in left and middle panel, and 0.5 mm in right panel. Experiment shown in panel A-C was repeated at least ten times. **d)** Leaves of five-week-old *rbohD* plants floating in water with undamaged leaf edge (left panels) or wounded leaf edge (right panels) were mock inoculated (10 mM MgCl_2_) or with *Xanthomonas* Leaf131 or Leaf148 (OD=0.02).

**Extended Data Fig. 3:**
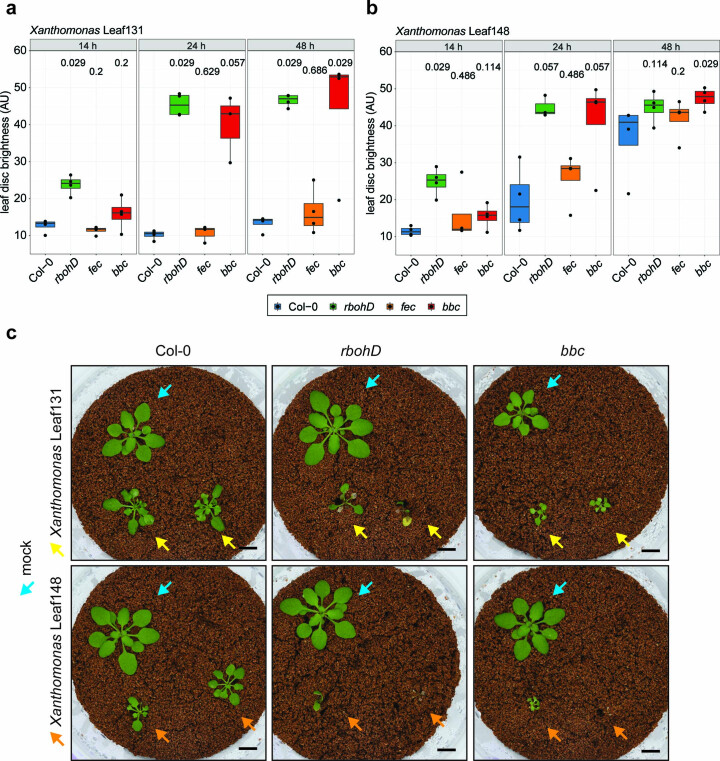
Plant genotype influences leaf disc degradation and susceptibility to *Xanthomonas*. **a)** Time-course of leaf discs brightness from six-week-old Col-0, *rbohD, fls2/efr/cerk1* (*fec*) and *bak1/bkk1/cerk1* (*bbc*) plants inoculated with *Xanthomonas* Leaf131 or **b)** Leaf148. Statistical differences of leaf disc brightness between plant genotype at varying time points is indicated with p-value above box plots (two-sided Mann–Whitney *U*-test, n = 4). Box plots show the median with upper and lower quartiles and whiskers present 1.5x interquartile range. **c)** Plant phenotype of gnotobiotic Col-0, *rbohD* and *bbc* plants mock inoculated (blue arrow) or with *Xanthomonas* Leaf131 (yellow arrow) or *Xanthomonas* Leaf148 (orange arrow) at 24 days post inoculation. Source data

Leaf tissue degradation progressed gradually over time starting at the edges of the leaf discs (Fig. 2b and Extended Data Fig. 2a). After complete degradation of leaf tissue, the leaf disc was translucent and eventually lost its cellular cohesion and fragmented after mechanical impact (Fig. 2c and Extended Data Fig. 2b). In contrast to the effective degradation of leaf discs from *rbohD* plants, those from Col-0 plants showed reduced and patchy degradation even after 48 h (Extended Data Fig. 2c). We tested other plant genotypes impaired in pattern-triggered immunity signalling upstream of RBOHD, such as hyper-susceptible mutants lacking cell surface localized receptors (for example, Flagellin Sensitive 2 (FLS2)) or mutants of co-receptors (for example, BRI1-Associated Receptor Kinase 1 (BAK1))^30,31^. We found that the triple co-receptor mutant *bak1*/*bkk1*/*cerk1* (*bbc*) but not the triple receptor mutant *fls2*/*efr*/*cerk1* was susceptible to leaf disc degradation similar to *rbohD* (Extended Data Fig. 3a, b). Consistently, *bbc* plants showed disease symptoms and reduced growth after inoculation with *Xanthomonas* Leaf131 and Leaf148 (Extended Data Fig. 3c). Besides plant genotype, plant age influenced leaf disc degradation, with 5-week-old Col-0 plants being more susceptible compared with 6-week-old plants (Supplementary Fig. 1), suggesting that multiple plant factors affect the phenotype. While we found that intact leaves remained visually unaffected upon exposure to *Xanthomonas* Leaf131 and Leaf148 within 2 days of observation, wounded leaves showed signs of degradation over the same time period, as expected due to more accessible tissue (Extended Data Fig. 2d). *Xanthomonas* Leaf131 caused disease and stunted plant growth in germ-free *rbohD* not only upon inoculation of 10-day-old seedlings^9^, but also resulted in disease symptoms and reduced growth in older *rbohD* plants after spray inoculation (Supplementary Fig. 2a,b). Despite *rbohD*-dependent disease symptoms, bacterial colonization was not significantly different between genotypes Col-0 and *rbohD* (Supplementary Fig. 2c,d). Spray inoculation of 5-week-old microbiota-free *rbohD* plants with *Xanthomonas* Leaf131 Tn7::Gm-lux led to disease symptoms 2 days after infection and co-localized with bacterial colonization based on luminescence (Supplementary Fig. 2e).

In general, our data indicate that opportunistic *Xanthomonas* spp. act as commensals in Col-0 plants and reveal their pathogenic potential in immunocompromised mutant plants where they elicit strong disease symptoms, in particular in the absence of a microbiota.

## Secretion of cell wall-degrading enzymes via T2SS Xps

**Fig. 3:**
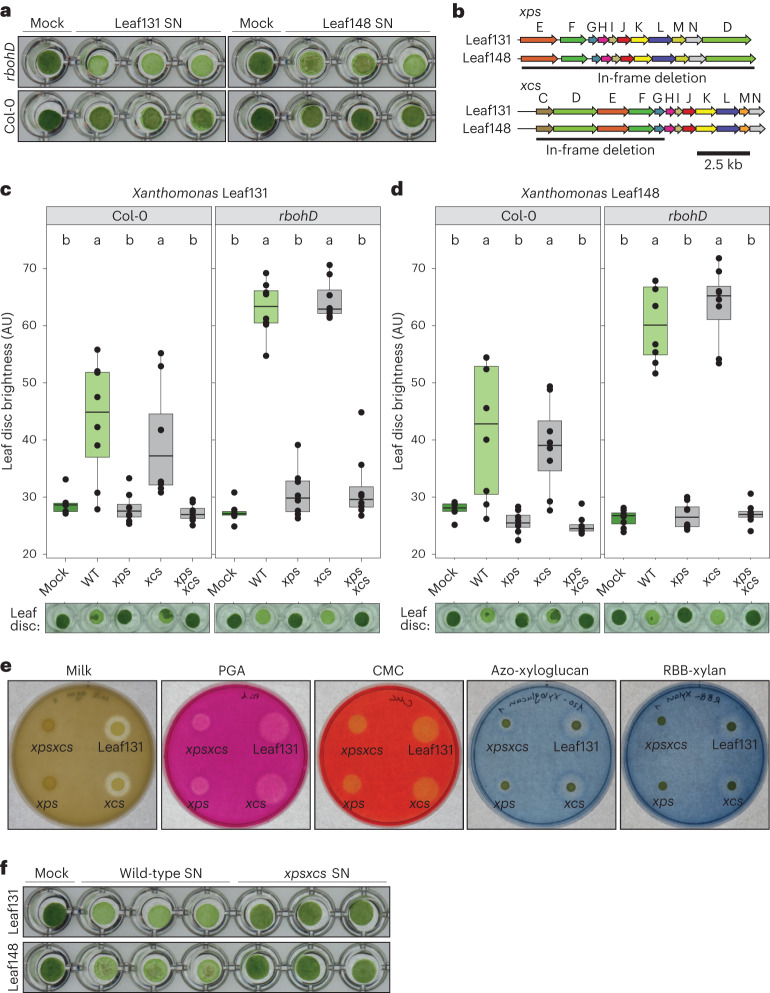
T2SS Xps requirement for leaf tissue degradation and secretion of plant polymer-degradative enzymes. **a**, Leaf discs of Col-0 and *rbohD* plants (5 weeks old) were mock treated (0.5× LB) or treated with cell-free supernatant (0.22 µm filter sterilized) of *Xanthomonas* Leaf131 or Leaf148 liquid cultures and incubated for 48 h. **b**, Genomic region of the T2SS operons *xps* and *xcs* in *Xanthomonas* Leaf131 and Leaf148. Letters indicate gene names and black line shows region of gene deletion. **c**,**d**, Leaf disc brightness was measured 24 h after inoculation with mock solution or with *Xanthomonas* wild-type or mutant strains of Leaf131 (**c**) or Leaf148 (**d**). Leaf discs were generated from Col-0 or *rbohD* plants (6 weeks old). Box plots show the median with upper and lower quartiles and whiskers present 1.5× interquartile range. Significant differences were calculated with ANOVA and two-sided Tukey’s honest significant difference post hoc test (*n* = 8, letters indicate significance groups, *α* = 0.05). **e**, Agar plates containing either skimmed milk, PGA, CMC, azo-xyloglucan or RBB-Xylan. Drops of 4 µl *Xanthomonas* Leaf131 wild-type or mutant suspension were pipetted onto agar plate. Pictures were taken 24 h after incubation at 22 °C. Quantification of halo diameter is shown in Supplementary Fig. 4. **f**, Leaf discs were treated with 0.22 µm filter-sterilized supernatant of liquid cultures from *Xanthomonas* Leaf131 or Leaf148 wild-type and *xpsxcs* mutants or mock solution (0.5× LB). Leaf discs were incubated for 48 h at 22 °C. AU, arbitrary unit; SN, supernatant; WT, wild type. Source data

Leaf tissue degradation by *Xanthomonas* as a proxy for a virulence phenotype was observed by live bacteria but also by cell-free supernatants of liquid cultures (Fig. 3a), indicating that the phenotype is mediated by secreted factors. Consistent with this finding, the secretion of plant cell wall-degrading enzymes (CWDE) by the T2SS is a known virulence function of other *Xanthomonas* species^32,33^. *Xanthomonas* Leaf131 and Leaf148 each possess two T2SS gene clusters, designated *xps* and *xcs* by homology search. To test whether the degradation activity is dependent on the T2SS, we deleted the core genes of the two T2SS operons (Fig. 3b) and generated double mutants in *Xanthomonas* Leaf131 and Leaf148. In both strains, the *xps* mutant and the double knockout *xpsxcs* did not show tissue degradation, in contrast to the *xcs* mutants, which were still able to degrade leaf discs (Fig. 3c,d). This indicates that the T2SS Xps is required for leaf degradation by *Xanthomonas*, which is in line with studies of other *Xanthomonas* bacteria reporting the importance of *xps* for virulence^34–36^.

In addition, we deleted the *hrpX* and *hrpG* genes in *Xanthomonas* Leaf131, which encode master regulators of virulence factors including T2SS-secreted enzymes in various *Xanthomonas* pathogens^37–41^. However, the *hrpXhrpG* double knockout mutant still showed leaf degradation activity (Supplementary Fig. 3) suggesting that the production or secretion of the degradative enzymes is not, or not exclusively, controlled by HrpX or HrpG in *Xanthomonas* Leaf131. In line with the absence of a phenotype for the *hrpXhrpG* knockout, transcriptomic studies in *Xanthomonas campestris* pv. *campestris* found that the T2SS genes and some (but not all) T2SS substrates were regulated by HrpG and that only a small subset of all genes regulated *in planta* were part of the HrpG regulon^42^.

To validate the finding that Xps is the primary T2SS involved in the secretion of plant polymer-degrading enzymes, we conducted agar plate assays using substrates for CWDE. We tested *Xanthomonas* Leaf131 and found that the strain was able to degrade milk powder, polygalacturonic acid (PGA), carboxymethyl cellulose (CMC), xyloglucan and xylan, suggesting secretion of proteases, pectate lyases, glucanases and xylanases, respectively, as shown by halos that formed around the bacterial colonies after incubation indicating substrate degradation (Fig. 3e). Notably, the T2SS mutants *xps* and *xpsxcs* showed reduced or delayed polymer degradation, unlike the *xcs* mutant strain (Fig. 3e). However, *xps* and *xpsxcs* mutants still resulted in a small halo indicating substrate degradation on xyloglucan plates after 24 h of incubation. At later timepoints, halos were observable on all plates (Supplementary Fig. 4), which might be due to cell lysis or alternative secretion mechanisms, such as outer membrane vesicles^43^.

**Extended Data Fig. 4:**
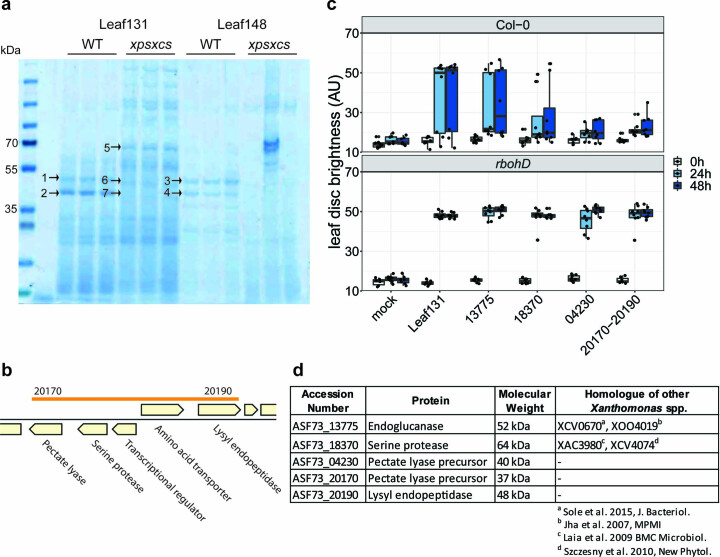
Proteomic analysis of supernatant from *Xanthomonas* Leaf131 and Leaf148 liquid culture identified T2SS-specific proteins. **a)** Coomassie stained SDS-PAGE of cell-free supernatant from *Xanthomonas* Leaf131 and Leaf148 wildtypes and *xpsxcs* mutants liquid cultures (n=3). Black arrows with numbers indicate protein bands excised from gel and analysed by LC-MS/MS with results of different fractions shown in Supplementary Table 2. **b)** Genomic region encoding T2SS-dependent secreted proteins pectate lyase (ASF73_20170) and lysyl endopeptidase (ASF73_20190). Orange line indicates in-frame deletion of gene cluster. **c)** Leaf discs of Col-0 or *rbohD* plants (six weeks old) were mock treated or with *Xanthomonas* Leaf131 wildtype or mutant strains with gene deletions of ASF73_13775, ASF73_18370, ASF73_04230, ASF73_20170-ASF73_20190. Box plots show the median with upper and lower quartiles and whiskers present 1.5x interquartile range. Sample size, n = 8. **d)** Table shows candidate genes of T2SS-dependent secreted proteins identified by LC-MS/MS (Supplementary Table 1) and gene identifiers of homologues in other *Xanthomonas* species as described in the literature. Source data

Next, we tested the leaf degradation activity of supernatants from *Xanthomonas* grown in liquid culture. In contrast to the wild type, cell-free supernatant of *Xanthomonas* Leaf131 and Leaf148 T2SS mutant *xpsxcs* did not cause *rbohD* leaf disc degradation (Fig. 3f). Sodium dodecyl sulfate–polyacrylamide gel electrophoresis analysis of supernatants revealed the presence of protein bands (35–55 kDa) in the wild type that were absent in the *xpsxcs* mutant (Extended Data Fig. 4a). Identification of the corresponding protein fractions by liquid chromatography tandem mass spectometry (LC–MS/MS) showed T2SS-dependent secretion (Supplementary Table 1) and several candidate proteins predicted to harbour a secretion signal peptide and a function potentially involved in plant interaction (Extended Data Fig. 4d). This included genes annotated to encode an endoglucanase (ASF73_13775), a serine protease (ASF73_18370), two pectate lyases (ASF73_04230 and ASF73_20170) and a lysyl endopeptidase (ASF73_20190), which is in line with the activities observed in the agar plate assays. We generated in-frame deletion knockout strains in *Xanthomonas* Leaf131 and tested the mutant strains for their leaf tissue degradation activity. For ASF73_20170 and ASF73_20190, which are located in proximity in the genome, we deleted the whole cluster (Extended Data Fig. 4b). Degradation was not affected in these mutant strains compared with wild type in *rbohD* leaf discs (Extended Data Fig. 4c). The mutant strains lacking a serine protease (ASF73_18370), a pectate lyase (ASF73_04230) or the gene cluster mutant ASF73_20170-20190 showed a difference in degradation in Col-0 (Extended Data Fig. 4c); however, this difference was not observed consistently, as leaf degradation in Col-0 is, in general, less pronounced, slower and more variable compared with *rbohD* (Fig. 2b and Extended Data Fig. 2c).

Overall, our data suggest that *Xanthomonas* secretes a cocktail of potential CWDE responsible for leaf degradation via the T2SS Xps.

## Involvement of T2SS Xps in virulence during plant infection

**Fig. 4:**
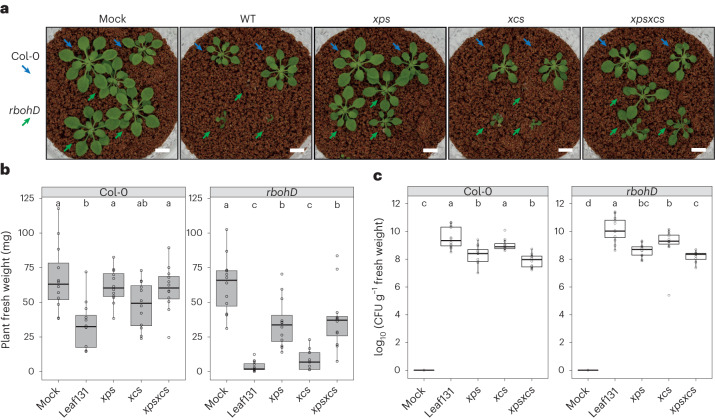
T2SS Xps requirement for full virulence and fitness of *Xanthomonas* Leaf131 *in planta*. **a**, Phenotype of 5-week-old Col-0 plants (blue arrow) and *rbohD* plants (green arrow) mock inoculated or with *Xanthomonas* Leaf131 wild type (WT) or T2SS mutants *xps*, *xcs* and *xpsxcs*. Scale bars, 1 cm. **b**, Measurement of fresh weight from plants shown in **a**. **c**, CFU counts of *Xanthomonas* Leaf131 per gram plant fresh weight from samples in **b**. Box plots show the median with upper and lower quartiles and whiskers present 1.5× interquartile range. Significant differences in **b** (*n* = 20) and **c** (*n* = 12) were calculated with ANOVA and two-sided Tukey’s honest significant difference post hoc test (letters indicate significance groups, *α* = 0.05). Log reduction of bacterial abundance shown in Source Data Fig. 4. Source data

To test the importance of the T2SS for virulence *in planta*, we inoculated Col-0 and *rbohD* plants with *Xanthomonas* Leaf131 wild type and the T2SS mutants. Plant health was monitored by assessing disease symptoms and measuring plant fresh weight 3 weeks after inoculation using an established gnotobiotic growth system^9^. The infection experiment revealed that the virulence of *Xanthomonas* Leaf131 was dependent on the presence of the T2SS Xps, while Xcs did not contribute to virulence (Fig. 4a, b), corroborating the results of the leaf degradation assay (Fig. 3c). While the *Xanthomonas* Leaf131 *xps* mutant was non-virulent in Col-0 plants, as indicated by similar plant weight compared with mock inoculation, the *xps* mutant showed residual virulence in *rbohD* plants (Fig. 4b), which suggests the presence of additional T2SS-independent virulence factors or alternative secretion pathways of leaf-degrading enzymes. Moreover, the overall colonization level of these disease-attenuated T2SS mutants *xps* and *xpsxcs* was significantly reduced by up to two orders of magnitude compared with *Xanthomonas* Leaf131 wild type in Col-0 and *rbohD* plants (Fig. 4c and Source Data for statistical results), highlighting the importance of the T2SS Xps for bacterial growth during plant colonization.

In addition to using a targeted approach by mutating the T2SS and genes for proteins that we found excreted under in vitro conditions, we used an untargeted approach by setting up a forward genetic screen in *Xanthomonas* Leaf131. The screening procedure was effective as we identified transposon (Tn) mutants that we had already confirmed as being important, that is the T2SS *xps*, and by identifying multiple independent transposon insertions in the same gene, suggesting high coverage (Supplementary Table 2). We identified 16 Tn mutant candidates with reduced or delayed leaf tissue degradation activity (Supplementary Note). To validate the results of the Tn screen, we generated and tested mutants in candidate genes by assessing leaf degradation phenotypes (Supplementary Fig. 5 and Supplementary Table 2) and virulence *in planta* (Supplementary Fig. 6). The selected targets were *dsbB* (ASF73_01480) encoding a thiol-disulfide interchange protein; *gtf*, encoding a predicted glycosyltransferase (ASF73_08425), located upstream of a flagellum gene cluster; and a gene (ASF73_19940) encoding for a hypothetical protein with glucanase/lectin domain. In addition, we deleted a gene cluster including the operon encoding the identified glucanase, a TonB-dependent receptor, a pectin methylesterase and a pectate lyase, as well as the TonB-dependent receptor, which is named *iroN* (ASF73_19920) and has been identified in the transposon screen (Supplementary Fig. 7).

**Fig. 5:**
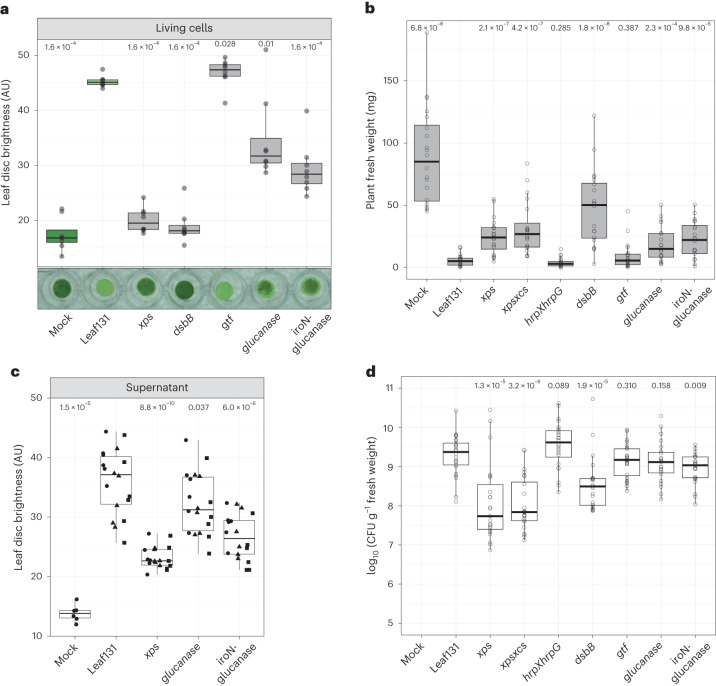
Additional virulence factors contribute to leaf degradation and virulence of *Xanthomonas* Leaf131. **a**, Leaf discs of 5-week-old *rbohD* plants were mock (10 mM MgCl_2_) inoculated or inoculated with *Xanthomonas* Leaf131 wild type (WT) or gene deletion mutants (OD of 0.02) and incubated for 24 h. **b**, Fresh weight of aboveground plant tissue of 5-week-old gnotobiotic *rbohD* plants, either mock inoculated or inoculated with *Xanthomonas* Leaf131 wild type or gene deletion mutants. **c**, Leaf discs of 5-week-old *rbohD* plants were mock treated (0.5× LB) or treated with cell-free supernatant (0.22 µm filter sterilized) of liquid cultures from *Xanthomonas* Leaf131 wild type and gene deletion mutants. Leaf discs were incubated for 24 h at 22 °C. Black circles, rectangles and squares indicate data from three bacterial cultures. **d**, CFU counts of *Xanthomonas* Leaf131 per gram plant fresh weight from samples in **b**. Box plots show the median with upper and lower quartiles and whiskers present 1.5× interquartile range. Significant difference in **a** (*n* = 8), **b** (*n* = 20), **c** (*n* = 24) and **d** (*n* = 12) of gene deletion mutants compared with Leaf131 wild type was determined by two-sided Mann–Whitney *U* test, and *P* values are indicated above box plots. Source data

We examined the ability of the gene deletion strains to degrade plant tissue and their impact on plant fresh weight during *rbohD* infection. With the exception of the *gtf* mutant strain, all other mutants showed phenotypes. Leaf degradation by the *dsbB* mutant was abolished in *rbohD*, similar to the *xps* mutant (Fig. 5a). In accordance with the impaired leaf degradation, the *dsbB* mutant was also reduced in virulence as indicated by higher fresh weight of *dsbB* colonized *rbohD* plants (Fig. 5b) and had a lower colonization level compared with the wild type, similar to *xps* and *xpsxcs* mutants (Fig. 5d). DsbB is involved in post-translational modification of secreted enzymes, including proteins of the T2SS, which therefore explains the similar phenotypes between the mutants^44^. The glucanase and *iroN*-glucanase mutants showed reduced or delayed leaf degradation in *rbohD* (Fig. 5a) and cell-free supernatant of liquid culture from the respective mutants revealed reduced degradation activities in *rbohD* leaf discs (Fig. 5c). This finding suggests that the glucanase might be directly involved in polymer degradation. All mutants with reduced degradation activity were also attenuated in overall virulence as indicated by higher fresh weight of *rbohD* plants (Fig. 5b), while glucanase and *iroN*-glucanase mutants maintained wild-type colonization levels (Fig. 5d). The gene encoding glucanase, which is absent in the glucanase and *iroN*-glucanase mutants (Supplementary Fig. 7), encodes a protein belonging to the glucanase superfamily (pfam13385) and contains a signal peptide for secretion. This glucanase contributed to leaf degradation and virulence *in planta*, which was notable given the functional redundancy common to tissue-degrading enzymes.

## *Xanthomonas* T2SS drives community shifts in *rbohD* plants

**Fig. 6:**
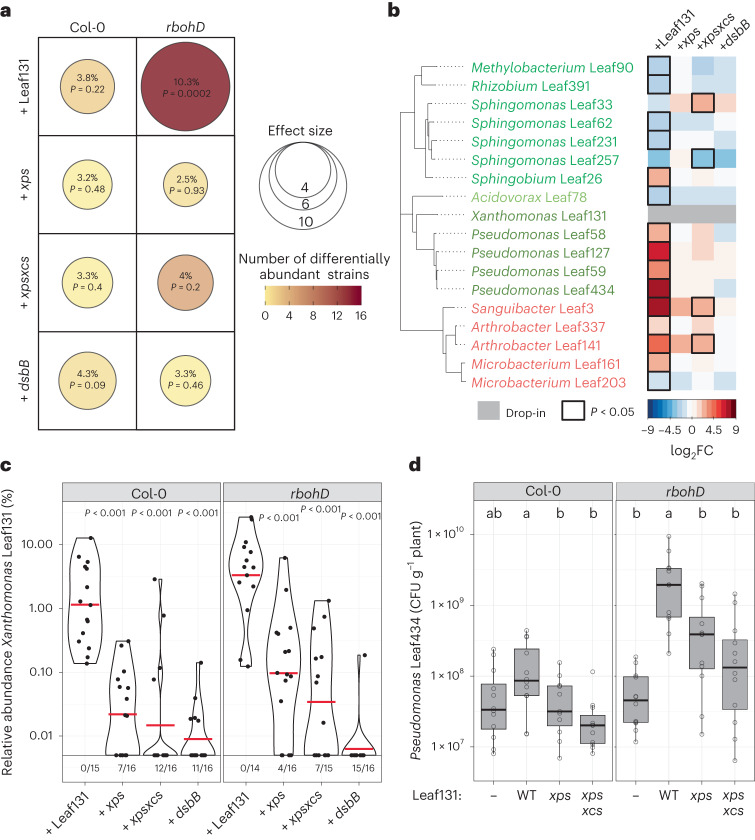
Microbiota shift in *rbohD* depends on T2SS-related virulence of *Xanthomonas* Leaf131. **a**, Composition of synthetic bacterial community SynCom-137 containing *Xanthomonas* Leaf131 wild type or mutants *xps*, *xpsxcs* and *dsbB* was compared with SynCom-137 alone in Col-0 and *rbohD* plants. Effect size represents percentage of total variance explained by genotype (shown by dot size and absolute value) and statistical significance is expressed with *P* values determined by PERMANOVA (Benjamini–Hochberg adjusted, *n* = 16). Number of differentially abundant strains (as shown in **b**) is represented by dot colour. **b**, Heatmap shows subset of strains of SynCom-137 with significant log_2_ fold changes (log_2_FC, *P* < 0.05) in *rbohD* plants inoculated either with only SynCom-137 or with SynCom-137 containing *Xanthomonas* Leaf131 wild type or the mutants *xps*, *xpsxcs* and *dsbB*. Black rectangles show significant changes, *P* < 0.05 (*n* = 16, two-sided Wald test, Benjamini–Hochberg adjusted). The heatmap of all strains in SynCom-137 is shown in Extended Data Fig. 5b. **c**, Relative abundance of *Xanthomonas* Leaf131 wild type or the mutants *xps*, *xpsxcs* and *dsbB* within SynCom-137 in Col-0 and *rbohD* plants. Ratios below violin plots represent frequency of samples where *Xanthomonas* Leaf131 was not detected. Violin plots show the median with upper and lower quartiles (*n* = 16, two-sided Mann–Whitney *U* test; *P* values are indicated above violin plots). **d**, CFU counts of *Pseudomonas* Leaf434 per gram plant fresh weight after inoculation of germ-free Col-0 and *rbohD* plants with *Pseudomonas* Leaf434 as single inoculation (−) or as binary inoculation with either *Xanthomonas* Leaf131 wild type or the mutants *xps* and *xpsxcs*. Box plots show the median with upper and lower quartiles and whiskers present 1.5× interquartile range. Significant differences were calculated with ANOVA and two-sided Tukey’s honest significant difference post hoc test (*n* = 12, letters indicate significance groups, *α* = 0.05). Source data

**Extended Data Fig. 5:**
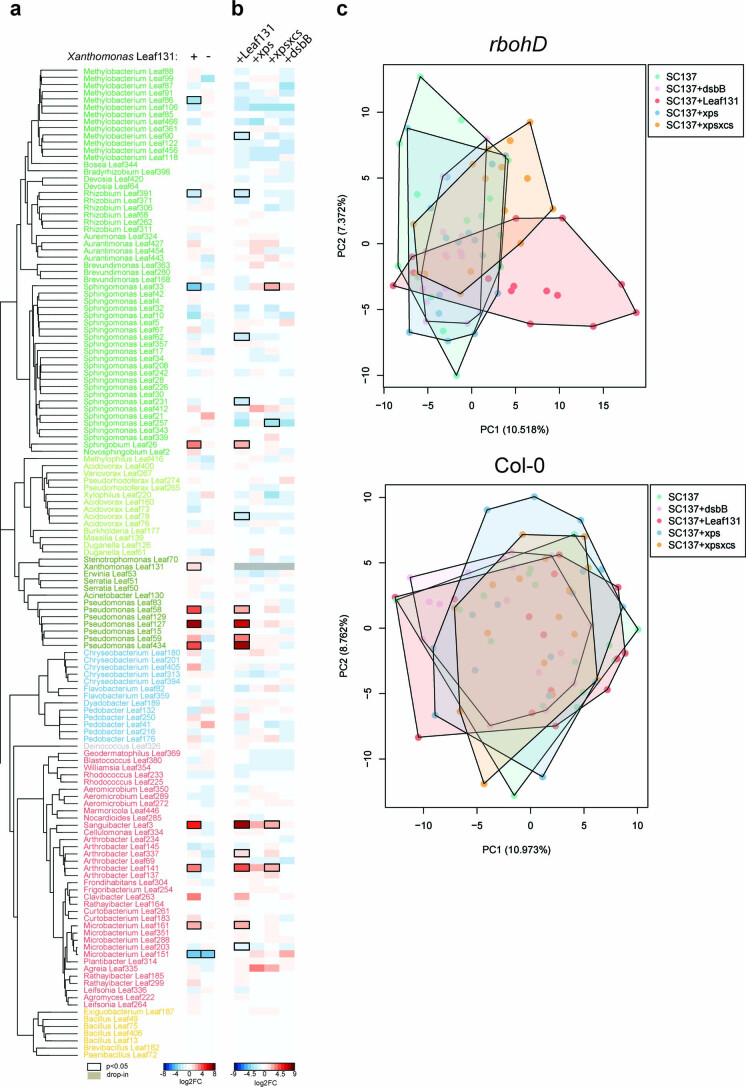
Microbiota composition in presence of virulent or attenuated *Xanthomonas* Leaf131. **a)** Heatmap shows log_2_ fold changes (log2FC) of strains in SynCom-137 in *rbohD* compared to Col-0 wild-type plants in the presence (+) or absence (−) of *Xanthomonas* Leaf131. **b)** Heatmap shows log_2_ fold changes (log2FC) of strains in the presence of either *Xanthomonas* Leaf131 wildtype or the mutants *xps*, *xpsxcs*, or *dsbB* compared to SynCom-137 without Leaf131. Black rectangles show significant changes, p-value < 0.05 (n = 16, two-sided Wald test, Benjamini–Hochberg adjusted). Subset of same data is shown in Fig. 6a. **c)** Principal component analysis of community in *rbohD* plants (upper panel) and Col-0 (lower panel) inoculated only with SynCom-137 or SynCom-137 containing either *Xanthomonas* Leaf131, *xps*, *xpsxcs*, or *dsbB*. Axes show principal components PC1 and PC2 with their explained variance (%). Source data

The secretion of extracellular enzymes is a crucial virulence factor of opportunistic *Xanthomonas* bacteria for plant colonization (Figs. 4 and 5), and our SynCom experiments revealed that plant disease and the microbiota shift in *rbohD* depend on the presence of *Xanthomonas* Leaf131 (Fig. 1). To investigate whether both phenotypes are causally linked and dependent on T2SS-related virulence, we inoculated plants with the SynCom-137 and added either *Xanthomonas* Leaf131 wild-type or attenuated mutant strains. We determined the microbiota profiles by 16S rRNA amplicon sequencing and compared the community composition of the SynCom-137 containing *Xanthomonas* Leaf131 wild type or mutants with the SynCom-137 without Leaf131 as a control. Quantification of the impact of each *Xanthomonas* strain on the community composition revealed a significant effect only of *Xanthomonas* Leaf131 wild type (effect size 10.3%, *P* = 0.0002), as observed previously (Fig. 1a), but not the attenuated mutants in *rbohD* plants (Fig. 6a). Consistently, only the presence of virulent *Xanthomonas* Leaf131, but not mutants with defective T2SS or *dsbB* knockout, increased the relative abundance of other commensals (Fig. 6b). The addition of *Xanthomonas* Leaf131 wild type to the SynCom-137 showed the characteristic shift in specific strains (Fig. 6b and Extended Data Fig. 5a), as observed previously (Fig. 1b). In contrast, inoculation of *rbohD* plants with SynCom-137 containing the *Xanthomonas* Leaf131 mutants *xps*, *xpsxcs* or *dsbB* resulted in a similar overall community composition as the SynCom-137 alone in *rbohD* and in Col-0 plants, as indicated by few changes of individual strains in their relative abundance (Fig. 6b and Extended Data Fig. 5b) and by overlapping clusters of the different conditions in a PCA (Extended Data Fig. 5c). In addition, the T2SS mutants showed reduced relative abundance, and *dsbB* was hardly detected by 16S rRNA amplicon sequencing (Fig. 6c), which underlines the importance of these features for the competitiveness of *Xanthomonas* in the context of a bacterial community, similar to the plant inoculations with only *Xanthomonas* Leaf131 (Fig. 4c).

Furthermore, we examined in a binary strain inoculation experiment the colonization level of the commensal *Pseudomonas* Leaf434 in response to attenuated *Xanthomonas* Leaf131. Strikingly, the increase in the commensal *Pseudomonas* Leaf434 observed during co-inoculation with virulent *Xanthomonas* Leaf131 was significantly reduced when the T2SS mutants *xps* and *xpsxcs* were paired with the *Pseudomonas* strain (Fig. 6d). This finding supports the conclusion that commensals are enriched in their abundance in plants due to the virulence of *Xanthomonas* Leaf131, which is particularly pronounced in immunocompromised *rbohD* plants.

In summary, our results indicate that specific microbiota members benefit indirectly from *Xanthomonas* Leaf131 due to T2SS-dependent virulence causing plant disease, rather than from the presence of *Xanthomonas* Leaf131 or the knockout of RBOHD per se.

## Discussion

Dysbiosis is considered a condition with distorted microbiota with various compositional states, but associated to disease and often characterized by weakening of host control over microbial growth^45,46^. However, the concept of dysbiosis is controversial because the causal relationships are often unclear, that is whether the observed changes in the microbiota are caused by the host genotype or by infection with a pathogen, and whether a shift in the microbial composition is the consequence of host disease or promoting disease^4,47^. Several studies have reported dysbiosis in the phyllosphere of plants infected with a pathogen^48–52^; however, it remains to be shown whether the pathogen invaded the microbial community as external agent or was part of the microbiota that was initially kept under control. Indeed, environmental conditions and protective microbiota members determine the virulence of pathogens^28,30,53–55^. Recently, experimental studies described that a functional immune system is required to maintain microbiota homoeostasis and prevent dysbiosis^9,10,56^.

Our previous finding that *A. thaliana rbohD* mutants display a microbiota shift and the identification of *Xanthomonas* Leaf131 and Leaf148 as opportunistic pathogens^9^ gave us the opportunity to disentangle the causation of dysbiosis in a representative leaf microbiome context. We found that conditional pathogenicity of the microbiota member *Xanthomonas* Leaf131 in immunocompromised *rbohD* mutants is governed by the T2SS and results in a dysbiotic microbial community characterized by increased abundance of *Xanthomonas* Leaf131 and other strains (Figs. 1 and 6). The enriched commensals might benefit from nutrients released from the plant as ‘public good’ and depend on their metabolic capacity that shapes microbiota composition^57–59^.

We found that leaf tissue damage caused by secreted CWDE via the T2SS Xps is a major virulence strategy of *Xanthomonas* Leaf131 and Leaf148 during infection of *A. thaliana*. However, it is still unclear what underlies the context-dependent pathogenicity of *Xanthomonas* Leaf131 and Leaf148 in *rbohD* plants. Pathogenicity of these opportunistic *Xanthomonas* strains could be regulated by *rbohD*-specific cues (for example, nutrients, signalling molecules or absence of ROS) that trigger a behavioural switch in *Xanthomonas* towards a pathogenic lifestyle. It was recently shown in *Xanthomonas* *citri* pv. *citri* that degradation products from the plant cell wall polymer xyloglucan induce transcription of virulence factors^60^. In our leaf degradation experiments using supernatants, *Xanthomonas* produced CWDE during incubation in rich media, suggesting that CWDE production is a constitutive trait and might not be dependent on host signals. A complementary study on *Xanthomonas* Leaf148 confirmed the T2SS-dependent pathogenicity, and a bacterial transcriptomics experiment indicates that genes encoding the T2SS and CWDE are more highly expressed in *rbohD* knockout plants^61^. We identified several T2SS-dependent proteins to be secreted by *Xanthomonas* Leaf131 and Leaf148 in liquid culture. Notably, some of these have been described in the context of virulence of *Xanthomonas* pathogens^34,36,43,62^. We have not observed a reduction in leaf tissue degradation in the gene knockout strains for two of the identified enzymes, an endoglucanase or serine protease, which is, however, not surprising given that a deletion of a single T2SS substrate often does not show a phenotype presumably due to functional redundancy among the secreted proteins^34,36^.

Context-dependent pathogenicity of opportunistic *Xanthomonas* strains might rely on plant susceptibility due to altered immune signalling or physical barriers. The plant immune system detects microbial activity and monitors the cell wall integrity^63,64^. Loss of microbe/danger-associated molecular patterns-induced ROS production by RBOHD results in impaired immune signalling and increased susceptibility to bacterial and fungal pathogens^17,65,66^ as well as reduced cell wall remodelling and lignification^19,20,67^. To explain susceptibility to opportunistic *Xanthomonas*, *rbohD* plants could mount an insufficient defence response. Many pathogens secrete CWDE to degrade plant polymers at certain stages during the infection process^64,68^ and, in turn, defects in cell wall composition make plants more susceptible^69^. As such, *rbohD* plants might have cell wall defects due to altered polymer crosslinking, which is in accordance with our data showing that tissue degradation activity of cell-free supernatants is higher in *rbohD* compared with Col-0 leaf discs. In that case, opportunistic *Xanthomonas* would secrete CWDE that break down a vulnerable (pre-formed) cell wall of *rbohD* plants. Strikingly, we have identified a single gene (ASF73_19940), which is required for full leaf tissue degradation and virulence in *rbohD* plants, and encodes a protein annotated with a secretion signal and a glucanase/concanavalin A-like lectin domain, which is potentially involved in carbohydrate processing or adhesion. Importantly, in *A. thaliana* wild-type plants, both *Xanthomonas* Leaf131 and Leaf148 protect from the virulent pathogen *Pseudomonas syringae* pv. *syringae* DC300053 and the related *Xanthomonas* WCS2014-23 is enriched in *A. thaliana* plants and limits infections with *Hyaloperonospora arabidopsidis*^70,71^ highlighting that these *Xanthomonas* can also be advantageous for the host when their pathogenicity is constrained. Mammalian NADPH oxidases produce ROS as a cell-to-cell messenger regulating the intestinal barrier, which is required for microbiota homoeostasis^72^, and ROS also form a physical barrier, which is thought to keep certain bacteria at distance from the epithelial surface^24,73^. This draws attention to striking similarities in the molecular mechanisms for host control of microbiota homoeostasis across animal and plant kingdoms.

In conclusion, our study revealed the importance of the T2SS for opportunistic *Xanthomonas* strains both for their interaction with the plant and for their competitiveness within the microbiota. The conditional pathogenicity of this opportunistic microbiota member depends on the host genotype and impacts both plant health and the microbial community. Our findings establish a causal link between a single plant gene to a specific genus of bacteria that drives a microbiota shift and highlight the crucial role of opportunistic pathogens in dysbiosis.

## Methods

### Plant growth conditions in soil

*A. thaliana* wild-type Col-0, *bbc*^30^, *fls2/efr/cerk1*^31^, *rbohD* knockout mutant^17^ and complementation line *rbohD/pRBOHD::RBOHD-FLAG* (*rbohD/RBOHD*)^65^ were used in this study.

*A. thaliana* plants for leaf degradation assays were grown in peat-based potting soil (substrate 1, Klasmann-Deilmann) in a growth chamber (CU-41L4, Percival) under controlled conditions (11 h light cycle, 22 °C, 65% relative humidity, light intensity (photosynthetic active radiation) 200 µmol s^−1^ cm^−2^). Seeds were treated with 70% ethanol for 2 min, sown on soil and stratified for 2 days at 4 °C in the dark.

### Gnotobiotic plant growth and bacterial inoculation

Gnotobiotic plants were prepared and grown in sterile microboxes filled with calcined clay as described previously^9^.

For the SynCom, 138 strains were selected on the basis of the *At*-LSPHERE strain collection (Supplementary Table 3) to have maximal phylogenetic diversity and to distinguish all strains with 100% sequence identity representing amplicon sequence variants (ASVs)^9^. *Xanthomonas* Leaf131 was used as single inoculum or mixed into the SynCom-137.

Bacterial growth, mixing of the synthetic community and plant inoculation were done as described before^9^. Each strain was mixed in equal volume ratio for inoculum mix. Germ-free, 11-day-old seedlings were inoculated with 200 µl bacterial solution. Plants were harvested between 35 and 38 days after germination. Experiments with SynCom, single strain or binary strain inocula were done in the same procedure. Axenic plants in gnotobiotic system were inoculated with buffer only and used as control for contamination by plating plant homogenate to monitor bacterial growth and were included as negative control in 16S rRNA amplicon sequencing. To extract DNA for 16S rRNA amplicon sequencing, the phyllosphere was harvested, weighed and stored at −80 °C.

Spray inoculation was done with sterilized glass sprayer in 24-day-old or 38-day-old gnotobiotic plants with bacterial culture diluted in 10 mM MgCl_2_ to optical density (OD)_600_ of 0.2 or 0.001, as indicated in corresponding figure legend.

To determine bacterial colonization levels, the phyllosphere was harvested, weighed and homogenized in 10 mM MgCl_2_ and a dilution series plated on R2A and methanol (MeOH) agar plates to count colony forming units (CFUs) after 2 days incubation at 28 °C. We excluded completely necrotic or dead plants from CFU count analysis as this would introduce inaccuracies depending on the time passed between plant death and the sampling timepoint. In the binary plant colonization experiments, the dilution series was plated on R2A-MeOH agar plates containing 10 µg ml^−1^ gentamycin and 25 µg ml^−1^ chloramphenicol to select for *Xanthomonas* Leaf131 Tn7::Gm-lux and *Pseudomonas* Leaf434, respectively. In addition, *Xanthomonas* Leaf131 and *Pseudomonas* Leaf434 can be distinguished by yellow and white colony pigmentation, respectively.

Bacterial luminescence of *Xanthomonas* Leaf131 Tn7::Gm-lux was measured in planta using IVIS spectrum imaging system (Xenogen). Exposure was set to 50 s and emission filter to 500. Radiance values (p^−1^ s^−1^ cm^−2^ sr^−1^) were extracted and normalized to plant size by adjusting elliptic region of interest for each plant.

### 16S rRNA amplicon sequencing and analysis

DNA extraction and 16S rRNA amplicon sequencing was done as previously published^9^, but polymerase chain reaction (PCR) reactions for 16S rRNA amplification and barcoding were not done in technical triplicate here.

16S rRNA amplicon data processing was done as described previously^9,26^. The ASV table (Supplementary Table 3) of each experiment was processed in R v.3.6.3 as described previously^9^. To account for varying sequencing depths between samples, the ASV table was log normalized and variance stabilized by DESeq2 v.1.14.1. To examine the effect on individual strains between the test and control conditions, the output of DESeq2 provided log_2_ fold change values and strains were considered to be differentially abundant according to Wald test implemented in DESeq2. *P* values were adjusted for multiple testing using the Benjamini–Hochberg method implemented in DESeq2. The differential strain abundances between the test and control conditions were visualized as a heatmap. To assess the overall effect on communities, PCA was performed with the transformed ASV table using the prcomp command. The effect size represents the variance explained by the compared factor and was calculated on Euclidean distances followed by a permutational multivariate analysis of variance (PERMANOVA) to test for statistical significance using the adonis command of the package vegan v.2 v.5-4. To summarize the relative abundance of *Xanthomonas* Leaf131 in a sample, the relative abundance values were calculated by proportional normalization of each sample by its sequencing depth.

The following R packages were used during analysis and visualization: ape v.5.4 (ref. ^74^), ggplot2 v.3.3.0 (ref. ^75^), vegan v.2.5-4 (ref. ^76^), DESeq2 v.1.14.1 (ref. ^77^) and ggpubr v.0.3.0 (ref. ^78^).

### Leaf disc degradation assay

Leaf discs of 5- or 6-week-old *A. thaliana* plants grown in soil were collected using a 4-mm-diameter biopsy puncher (BPP-40F, KAI MEDICAL) and placed with the adaxial side up in a clear flat-bottom 96-well plate (655101, Greiner Bio-One) filled with 90 µl Milli-Q purified water. *Xanthomonas* were grown on R2A-MeOH agar plates for 2 days at 22 °C; bacterial cells were scraped off, resuspended in 10 mM MgCl_2_ by vortexing for 2 min and the bacterial solution was adjusted to OD_600_ of 0.1. Leaf discs were inoculated with 10 µl of bacterial suspension and incubated at 22 °C for up to 48 h in the dark. Digital images were taken at regular intervals under standardized conditions using a black box and a light screen illuminating leaf discs from below to monitor leaf tissue degradation.

### Quantification of leaf disc brightness

To quantify leaf tissue degradation, we developed a computational script MatlabR2022a (MathWorks), which recognizes leaf discs in a 96-well plate, measures surface area, brightness of the red channel (in RGB images) and computes a ‘roughness’ parameter.

In short: the script normalizes the brightness of the images using the ‘illumgray’ and ‘chromadapt’ functions implemented in MATLAB. Subsequently, a binary mask is created, separating the area occupied by leaf discs from the rest of the image. Discs that deviate in ‘roundness’ are discarded from the analysis since they are probably broken or folded. The roughness parameter is created using the ‘Sobel’ edge detection function on the isolated discs in the red channel and computing the total number of pixels recognized as edge within each disc. The area of each disc is computed by counting the number of pixels per disc times the pixel size retrieved from an image scaling step. The brightness value represents the mean brightness value of the red channel for each individual leaf disc.

The MatlabR2022a code and user manual is available^79^.

### Transformation of electrocompetent *Xanthomonas* cells

Electrocompetent *Xanthomonas* cells were made by an established protocol^80^. Exponentially growing *Xanthomonas* cells in 200 ml lysogeny broth (LB) at 28 °C with an OD_600_ between 0.6 and 1 were cooled on ice for 20 min and kept on ice for the entire procedure. Cells were collected by centrifugation for 15 min at 4,000*g* at 4 °C and washed three times in chilled sterile 10% glycerol to remove growth medium. After the final washing step, cells were concentrated approximately 100-fold compared with initial volume in 10% glycerol and aliquots frozen at −80 °C.

Electrocompetent *Xanthomonas* cells were thawed on ice and 50 µl was mixed with 200 ng plasmid. Cells were transformed by electroporation in 1-mm electro-cuvettes applying 1.8 kV electric current. Cells were recovered in LB medium for 2–4 h shaking at 28 °C before plating 100 µl on LB agar plates containing selective antibiotics.

*Xanthomonas* Leaf131 cells were transformed with pUC18-mini-Tn7T-Gm-lux^81^ and helper plasmid pTNS3 (ref. ^82^) for site-specific Tn7 integration of *luxCDABE*. Transformed cells of *Xanthomonas* Leaf131 Tn7::Gm-lux were selected on LB agar plates containing 10 µg ml^−1^ gentamycin.

### Bacterial gene knockout strains

Markerless gene deletion in *Xanthomonas* strains (Supplementary Table 4) were made according to a method based on double homologous recombination using the suicide plasmid pK18mobSacB as vector^83^. Gene deletion plasmids were designed to result in in-frame deletion of the gene of interest while leaving an open reading frame of three to four amino acid peptide. Briefly, 500 bp of flanking regions upstream and downstream of the gene of interest were amplified by PCR and cloned into pK18mobSacB plasmid. The plasmids were cloned using either classical restriction enzyme digest or Gibson Assembly (oligonucleotides are provided in Supplementary Table 4) in *Escherichia* *coli* DH5α. Gene deletion constructs were confirmed by Sanger sequencing.

Electrocompetent *Xanthomonas* cells were transformed, recovered in LB medium for 2–4 h and transformed cells were selected on LB agar plates containing 50 µg ml^−1^ kanamycin. Transformed cells were re-streaked on fresh selective LB agar plates and a single colony resuspended in LB medium for 2 h before plating on LB agar plates containing 5% sucrose to select for double cross-over events due to homologous recombination and chromosomal deletion of the gene of interest and the vector backbone. After sucrose selection, individual colonies were tested for sensitivity to kanamycin. Cells were re-streaked to obtain single colonies that were cultured and frozen in 25% glycerol at −80 °C. Genomic deletion was confirmed by PCR using primers outside of flanking regions and Sanger sequencing the PCR product and by the absence of PCR product using primers inside genomic deletion.

### Bacterial supernatant of liquid culture

*Xanthomonas* were grown in triplicates in 100 ml liquid 0.5× LB medium until late exponential growth phase (approximately OD_600_ of 2) at 28 °C while shaking. Cells were harvested by centrifugation at 4,000*g* for 15 min and washed twice in 10 mM MgCl_2_. Bacterial cells were resuspended in 10–20 ml fresh 0.5× LB medium at an OD_600_ of 3 and incubated for 4 h in flasks at 28 °C while shaking. To obtain the cell-free supernatant, we centrifuged the samples at 4,000*g* for 15 min to remove bacteria and filter sterilized the supernatant using 0.22-µm filter units (no. 99505, ‘rapid’-Filtermax, TTP) and a vacuum pump. A total of 10 ml of cell-free supernatant was concentrated ten-fold by using Ultrafiltration Units Amicon-15 with a molecular weight cutoff 10 kDa (Merck) and centrifugation at 3,500*g* at 4 °C for 20–40 min. Cell-free supernatants were directly tested for leaf degradation activity and kept on ice until further processing for protein analysis.

Cell-free supernatant or concentrated supernatant was applied to leaf discs to test for tissue degradation activity. Leaf discs were collected from 5- or 6-week-old plants and floated in 40 µl Milli-Q purified water in a 96-well plate. To each leaf disc, 40 µl supernatant was added. Leaf discs were incubated at 22 °C and photographs taken at regular intervals.

### Analysis of protein bands by LC–MS/MS

To test for the secretion of proteins, the concentrated cell-free supernatant of *Xanthomonas* liquid cultures was obtained as described above. Protein concentration of the concentrated supernatant was determined by the Pierce BCA assay kit (Thermo Fischer Scientific) according to the manufacturer’s instructions. Protein content of supernatant samples were normalized and analysed using sodium dodecyl sulfate–polyacrylamide gel electrophoresis (mPAGE Bis-Tris 8%, Merck) revealing specific protein bands in the supernatant while comparing wild type and the T2SS mutant (*xpsxcs*) of *Xanthomonas* Leaf131 and Leaf148. The protein bands of interest were cutout and identified by in-gel digestion and LC–MS/MS analysis as described previously^84^. Reference genomes of *Xanthomonas* Leaf131 and *Xanthomonas* Leaf148 accessed under NCBI:txid1736270 and NCBI:txid1736275, respectively.

### Substrate degradation by secreted enzymes in agar plates

Agar plate assays to detect glucanase, xylanase, pectate lyase and polygalacturonase or protease activity were modified after refs. ^85–87^.

*Xanthomonas* strains were streaked on R2A-MeOH plates and grown at 22 °C for 2 days. Bacterial cells were scraped off and resuspended in 1 ml 10 mM MgCl_2_ by vortexing for 5 min to disperse cell aggregates. Cell density was adjusted to OD_600_ of 0.4 and 4 µl of the bacterial suspensions were spotted on R2A agar plates either containing 0.5% sodium CMC (Sigma-Aldrich, C5678), 0.05% Remazol Brilliant Blue-Xylan (RBB-Xylan; Sigma-Aldrich, M5019), 0.1% azo-xyloglucan (Megazyme, S-AZXG) or 0.1% PGA in 1 M sodium phosphate buffer pH 7.0 (Sigma-Aldrich, 81325) or on 1.5% agar plates containing 3% skimmed milk powder (Rapilait), 1% peptone, 0.025% MgSO_4_ and 0.05% K_2_HPO_4_, respectively. The plates were incubated at 22 °C, and photographs were taken at regular intervals.

Glucanase activity can be detected by yellow halos against the red background after staining with 0.1% Congo red (Sigma-Aldrich, C6767) dye solution (solved in 50% ethanol) for 30 min and destaining with 1 M NaCl for 15 min. Pectate lyase or polygalacturonase activity can be detected by light-pink halos against the darker pink background after staining with 0.05% ruthenium red (Sigma-Aldrich, R2751) dye solution (solved in water) for 30 min and destaining with water. Xylanase or protease activity can be detected by a light-blue or clear halo forming around the colonies, respectively.

### Reporting summary

Further information on research design is available in the Nature Portfolio Reporting Summary linked to this article.

### Supplementary information

Supplementary Information Supplementary information.

Reporting Summary

Supplementary Tables 1–5 **Supplementary Table 1. Proteomics of Leaf131 culture supernatant. a**, Identification of proteins in fractions of *Xanthomonas* Leaf131 supernatant from wild type (fractions 1 and 2) or *xpsxcs* mutant (fractions 4 and 5). **b**, Identification of proteins in fractions of *Xanthomonas* Leaf148 supernatant from wild type (fractions 3 and 4). Fractions are protein bands excised from SDS–PAGE (Extended Data Fig. 4a). Selection for gene knockout highlighted in orange. **c**, Table presents selected protein candidates for gene knockout in *Xanthomonas* Leaf131. **Supplementary Table 2. Transposon mutagenesis screen in** ***Xanthomonas*** **Leaf131. a**, Overview of transposon screen and identified candidate genes. **b**, Selection of validated candidate genes. **Supplementary Table 3. SynCom strains and microbiota composition data. a**, *At*-LSPHERE strains used in SynCom-137 and *Xanthomonas* Leaf131 and Leaf148. **b**,**c**, ASV count table for drop-out experiment (**b**) and corresponding metadata (**c**). **d**,**e**, ASV count table for drop-in experiment (**d**) and corresponding metadata (**e**). **Supplementary Table 4. Knockout strains and oligonucleotides used in this study. Supplementary Table 5. Statistical analysis of data shown in Supplementary Fig. 1**. Results of two-way ANOVA of data shown in Supplementary Fig. 1.

### Source data

Source Data Fig. 1 Data tables for graphs and statistical analysis.

Source Data Fig. 2 Data tables for graphs and statistical analysis.

Source Data Fig. 3 Data tables for graphs and statistical analysis.

Source Data Fig. 4 Data tables for graphs and statistical analysis.

Source Data Fig. 5 Data tables for graphs and statistical analysis.

Source Data Fig. 6 Data tables for graphs and statistical analysis.

Source Data Extended Data Fig. 1 Data tables for graphs and statistical analysis.

Source Data Extended Data Fig. 3 Data tables for graphs and statistical analysis.

Source Data Extended Data Fig. 4 Data tables for graphs and statistical analysis.

Source Data Extended Data Fig. 5 Data tables for graphs and statistical analysis.

## Data Availability

Raw data of 16S rRNA amplicon sequencing can be found at the European Nucleotide Archive under accession number PRJEB64618. Source data are provided with this paper.

## Code availability

Customized code to analyse data and generate figures can be found at https://github.com/MicrobiologyETHZ/phylloR/releases/tag/v1.1. The code to quantify leaf disc brightness using MatlabR2022a and user manual is available at https://github.com/gaebeleinC/leaf-disc_quantification.
