# Supplementary material for: Leaf microbiome dysbiosis triggered by T2SS-dependent enzyme secretion from opportunistic Xanthomonas pathogens
Source: Nat Microbiol. 2024 Jan 3;9(1):136–49. doi: 10.1038/s41564-023-01555-z (PMC10769872; doi:10.1038/s41564-023-01555-z)
Supplement: Supplementary file 1 — Supplementary information. [file 41564_2023_1555_MOESM1_ESM.pdf]

nature microbiology

Article

https://doi.org/10.1038/s41564-023-01555-z

Leaf microbiome dysbiosis triggered by T2SS-dependent enzyme secretion from opportunistic *Xanthomonas* pathogens

In the format provided by the authors and unedited

Supplementary Note

Supplementary Figures 1-7

Leaf microbiome dysbiosis triggered by T2SS-dependent enzyme secretion from opportunistic *Xanthomonas* pathogens

Sebastian Pfeilmeier[1, 2]*, Anja Werz[1], Marine Ote[1], Miriam Bortfeld-Miller[1], Pascal Kirner[1], Andreas Keppler[1], Lucas Hemmerle[1], Christoph G. Gäbelein[1], Gabriella C. Petti[1], Sarah Wolf[1], Christine M. Pestalozzi[1], Julia A. Vorholt[1]*

[1]Institute of Microbiology, ETH Zurich, 8093 Zurich, Switzerland

[2]Molecular Plant Pathology, Swammerdam Institute of Life Sciences (SILS), University of Amsterdam, Amsterdam, Netherlands

*corresponding authors j.s.pfeilmeier@uva.nl, jvorholt@ethz.ch

Supplementary Note

Identification of virulence factors of *Xanthomonas* using a forward genetic screen

In addition to using a targeted approach by mutating T2SS and genes for proteins that we found excreted under *in vitro* conditions, we used an untargeted approach by setting up a forward genetic screen in *Xanthomonas* Leaf131. We used a vanillate-inducible, hyperactive himar transposase (pAK415-himar)[1] and tested more than 6000 transposon (Tn) mutants individually for their ability to degrade *rbohD* leaf discs. For all Tn mutants with delayed or impaired leaf degradation activity, we mapped the transposon insertion site by amplifying flanking regions using a nested-PCR approach with arbitrary PCR primers, followed by BLAST search with the sequenced PCR product[1]. After the primary screening, we validated 34 Tn mutant candidates on eight replicate leaf discs from *rbohD* plants to confirm their phenotypes and selected a subset of 16 Tn mutants based on their impairment in leaf disc degradation (Supplementary Figure 5 and Supplementary Table 2). Five Tn mutants lost leaf degrading capacity (Tn28_rplQ, Tn37_gtf, Tn68_dsbB, Tn80_xpsE, Tn81_xpsD) and five Tn mutants showed reduced leaf disc degradation (Tn10_glucanase, Tn15_iroN, Tn59_mopB, Tn69_flhA, Tn77_lolA) after 48 hours of co-incubation of bacteria and leaf discs, while six Tn mutants showed a delay of leaf disc degradation in *rbohD* only after 24 hours (Tn11_fliM, Tn13_tldD, Tn21_flgI, Tn24_dgkA, Tn26_XCC3185, Tn29_pilY) (Supplementary Figure 5). The screening procedure also resulted in the identification of the T2SS *xps*, and multiple independent transposon insertions in the same gene, suggesting high coverage (Supplementary Table 2).

The Tn mutants with strong impairment of leaf degradation at 48 hours encoded genes involved in protein secretion (Tn80_xpsE, Tn81_xpsD), motility (Tn69_flhA), post-translational protein modification (Tn68_dsbB), glycosylation (Tn37_gtf), predicted glucanase/lectin domain (Tn10_glucanase), transport (Tn15_iroN) and protein translation (Tn28_rplQ). In summary, the screen resulted in several promising candidate genes of *Xanthomonas* involved in leaf degradation.

In addition to the T2SS mutants, we tested the above identified Tn mutants for their virulence *in planta*. Seven out of 16 tested Tn mutants were less virulent than *Xanthomonas* Leaf131 wildtype during plant infection (Supplementary Figure 6b). A number of Tn mutants with defects in motility related genes (e.g. Tn21_flgI, Tn29_pilY, Tn11_filM, Tn69_flhA) had no significant impact on *rbohD* plant weight compared to *Xanthomonas* Leaf131 wildtype despite a delay in leaf tissue degradation (Supplementary Figure 5 and 6b). A potential explanation for this discrepancy could be that motility or adhesion to the leaf disc is critical for colonization or degradation activity up to 48 hours in liquid, but not relevant for virulence during plant colonization over a three-week-period. In addition, Tn mutants with reduced impact on plant weight also showed reduced absolute abundance on the host, as indicated by approx. 5-fold reduced cfu per gram fresh weight, similar as observed for the T2SS mutants (Supplementary Figure 6c).

Method of transposon mutagenesis screen

The transposon (Tn) mutagenesis screen was done by transforming electrocompetent cells of Xanthomonas Leaf131 with the pAK415 plasmid containing a vanillate-inducible, hyperactive transposase Himar1C9W[1]. After electroporation, bacteria were recovered in 500 µL LB containing 500 µM vanillate at 28°C shaking for 4 hours to induce temporal expression of the transposase.

Single colonies were selected on LB-agar plates containing 50 $\mu g*mL^{-1}$ kanamycin grown for two days at 28°C. Colonies were picked and grown in 100 µL liquid LB medium in 96-well plates. After measuring OD_{600} to monitor bacterial growth, each transposon mutant colony was tested for its potential

to degrade a leaf disc from five-week-old *A. thaliana rbohD* plants. Leaf disc degradation was scored manually at 24 and 48 hours. In total, 6016 mutants were screened and 92 Tn mutants showed either delayed or no leaf degradation after 24 hours (Supplementary Table 2). To validate the phenotype, we retested all identified Tn mutants on eight leaf discs of *rbohD* plants. For this, bacteria were grown on R2A-MeOH agar plates and resuspended in 10 mM $MgCl_2$ and leaf discs inoculated at an OD_{600} of 0.02. After the validation screening, 34 Tn mutants were confirmed to have a deficiency in *rbohD* leaf degradation after 48 hours.

To identify the Tn insertion site for each candidate Tn mutant, we mapped the genomic location by a nested semi-degenerated primer PCR approach[1,2]. The first PCR reaction was done with a mixture of semi-degenerated primers (Arb-P1, Arb-P2, Arb-P3) mixed in equal ratio and a transposon-specific primer (pAK411_nested1). In a 25 µL PCR reaction, 1 µL of 10 µM forward and reverse primers, 0.2 µL Phusion polymerase, 5 µL of 5x Phusion GC buffer, 1.25 µL dNTP mix, 0.75 µL DMSO and 1 µL of supernatant from heat-killed bacterial suspension were used as template. Hot-start PCR was done in a thermocycler using the following settings: initial 98°C for 3 min, 10 cycles of 98°C for 10 seconds, an annealing step starting at 35°C for 20 seconds and increasing 1°C in each cycle until 45°C, elongation at 72°C for 30 seconds; the temperature gradient cycles were followed by 25 cycles of 98°C for 10 seconds, annealing at 45°C for 20 seconds, elongation at 72°C for 30 seconds and a final elongation at 72°C for 5 minutes. The PCR product was visually inspected on an agarose gel and cleaned up using NucleoSpin gel and PCR clean-up kit (Machery-Nagel, Düren, Germany). The DNA fragments from the first PCR were used as templates in a second PCR using sequence-specific (nested) primer binding on the transposon and on a sequence introduced by the semi-degenerated primers. The PCR reaction was done in 50 µL total volume using 2 µL of 10 µM Anchor-P primer and pAK411_nested2 primer, 0.4 µL Phusion polymerase, 10 µL of 5x Phusion GC buffer, 2.5 µL dNTP mix, 1.5 µL DMSO and 1 µL of purified PCR product as template. Hot-start PCR was done with following settings: initial denaturing at 98°C for 3 min, 5 cycles of 98°C for 10 seconds, an annealing step starting at 63°C for 20 seconds and decreasing 1°C in each cycle until 59°C, elongation at 72°C for 30 seconds; the temperature gradient cycles were followed by 30 cycles of 98°C for 10 seconds, annealing at 58°C for 20 seconds, elongation at 72°C for 30 seconds and a final elongation at 72°C for 5 minutes. The product of the second PCR was cleaned-up and Sanger sequenced using primer pAK411_nested2. The genomic insertion site was identified by using the PCR amplified sequence in a BLAST search against the genome of *Xanthomonas* Leaf131 (NCBI:txid1736270) at NCBI and IMG/JGI.

Supplementary Figures

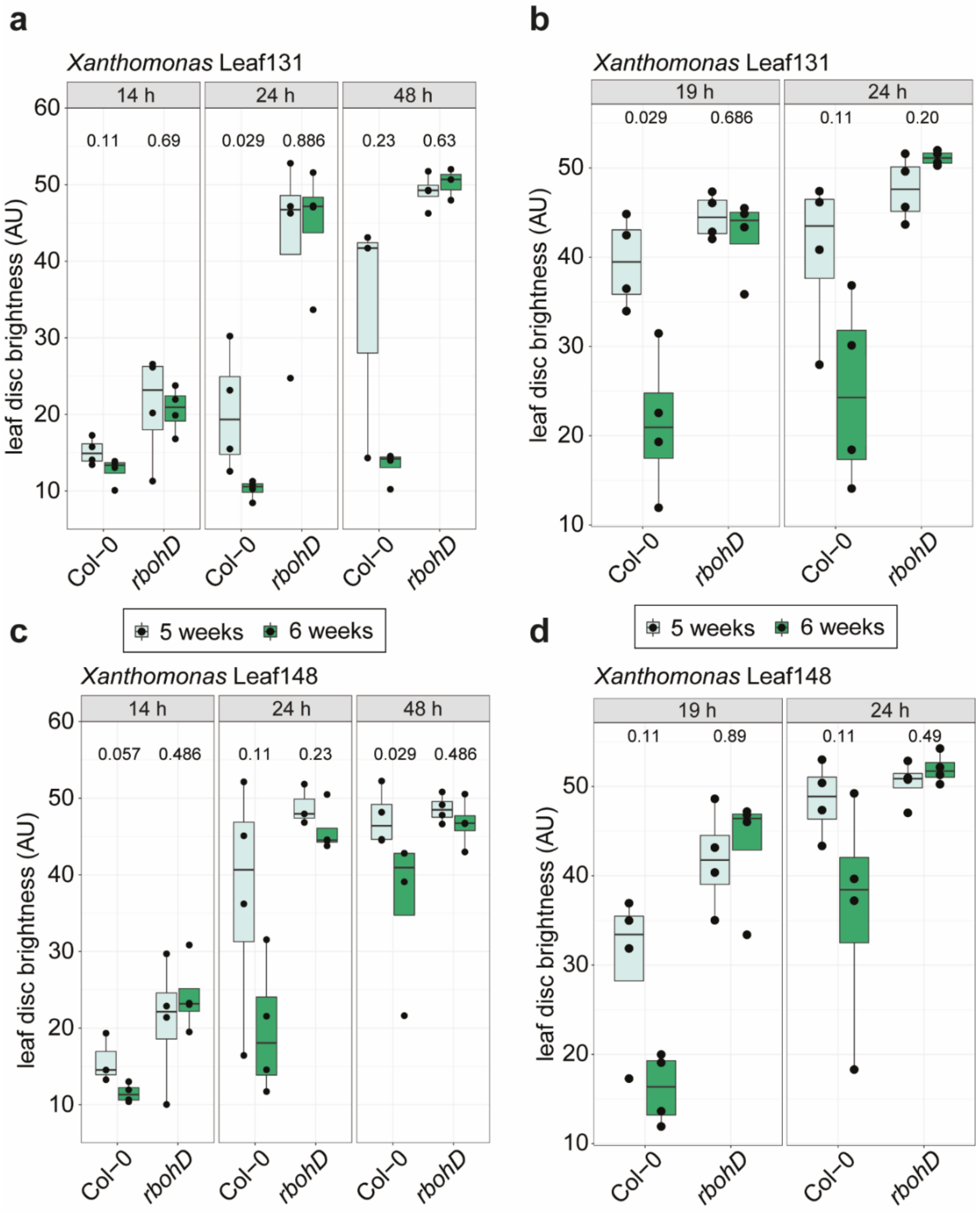

Supplementary Figure 1. Plant age influences leaf disc degradation by *Xanthomonas*. a) Time-course of leaf discs brightness from five- and six-week-old Col-0 and *rbohD* plants inoculated with *Xanthomonas* Leaf131 or **c)** Leaf148. **b)** and **d)** show independent replicate experiments for *Xanthomonas* Leaf131 and Leaf148, respectively. Statistical differences of leaf disc brightness between plant genotype at varying time points is indicated with p-value above box plots (two-sided Mann–Whitney U-test, n = 4). Box plots show the median with upper and lower quartiles and whiskers present 1.5x interquartile range. Two-way ANOVA of the data presented here show significant impact of genotype and plant age depending on time point (Supplementary Table 5).

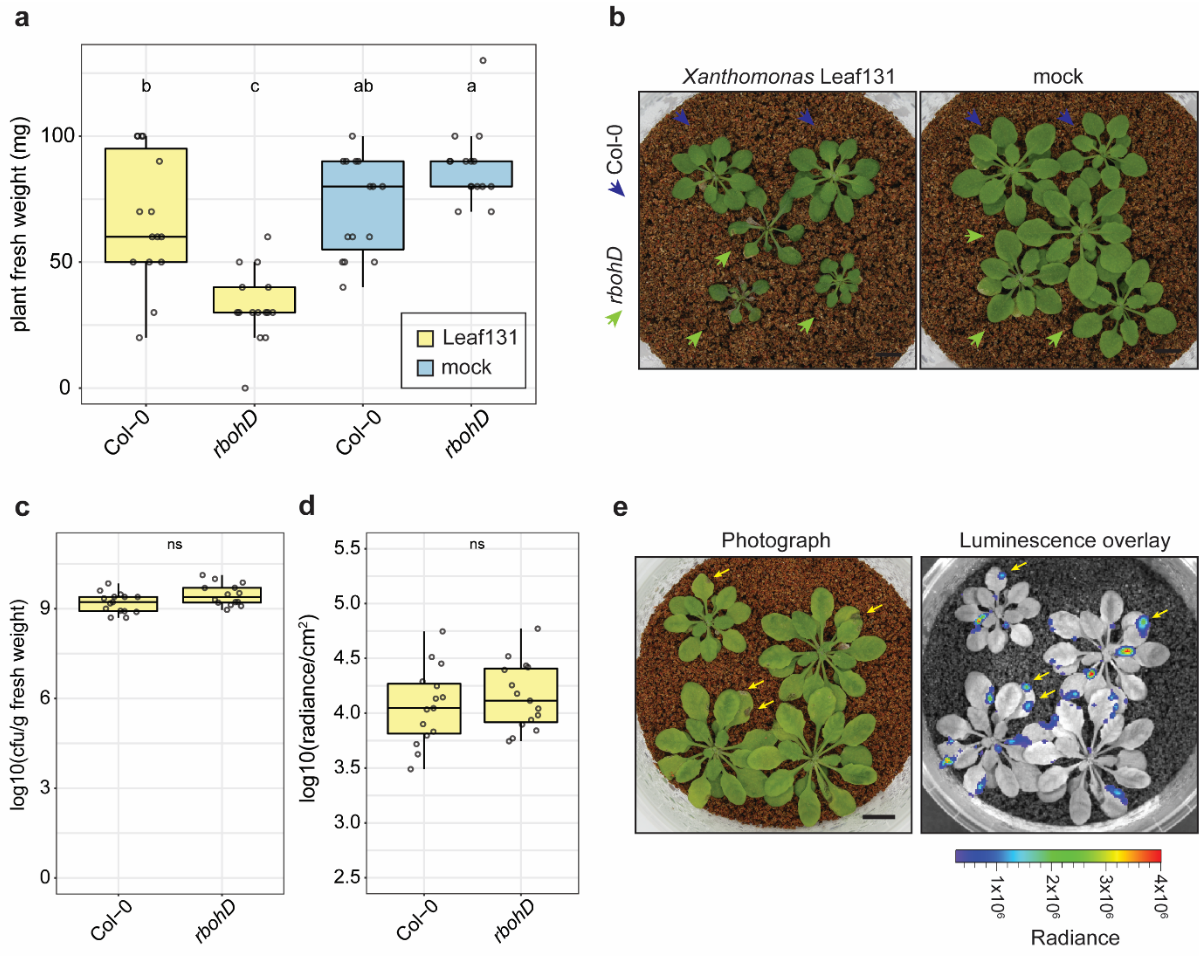

Supplementary Figure 2. *Xanthomonas* Leaf131 causes disease in *rbohD* plants after spray inoculation. a) Fresh weight of 38-days-old plants at 14 days after spray inoculation with 10 mM $MgCl_2$ (mock) or *Xanthomonas* Leaf131 Tn7::lux (OD_{600} = 0.2). **b)** Colony forming units (cfu) per gram fresh weight and **c)** luminescence measurements expressed as radiance (p/sec/cm^2/sr) of samples described in A). Box plots show the median with upper and lower quartiles and whiskers present 1.5× interquartile range. Significant differences were calculated with ANOVA and two-sided Tukey's HSD post hoc test (letters indicate significance groups, $\alpha = 0.05$, n=15). **d)** Microbiota-free *rbohD* plants (38-days-old) were spray-inoculated with *Xanthomonas* Leaf131 Tn7::lux (OD_{600} = 0.001). Photograph picture and luminescence overlay was taken two days post infection. Yellow arrows exemplarily highlight disease symptoms, which overlap with luminescent bacteria.

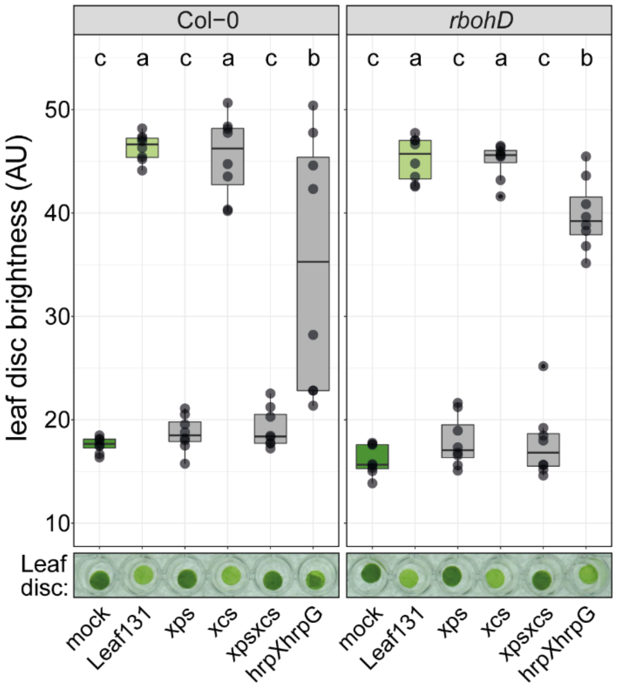

Supplementary Figure 3. *Xanthomonas* Leaf131 HrpX and HrpG are not necessary for leaf disc degradation. Leaf discs of Col-0 or *rbohD* plants (five weeks old) were mock treated or with *Xanthomonas* Leaf131 wildtype or mutant strains and incubated for 24 hours. Box plots show the median with upper and lower quartiles and whiskers present 1.5× interquartile range. Significant differences were calculated with ANOVA and two-sided Tukey's HSD post hoc test (n= 8, letters indicate significance groups, $\alpha = 0.05$).

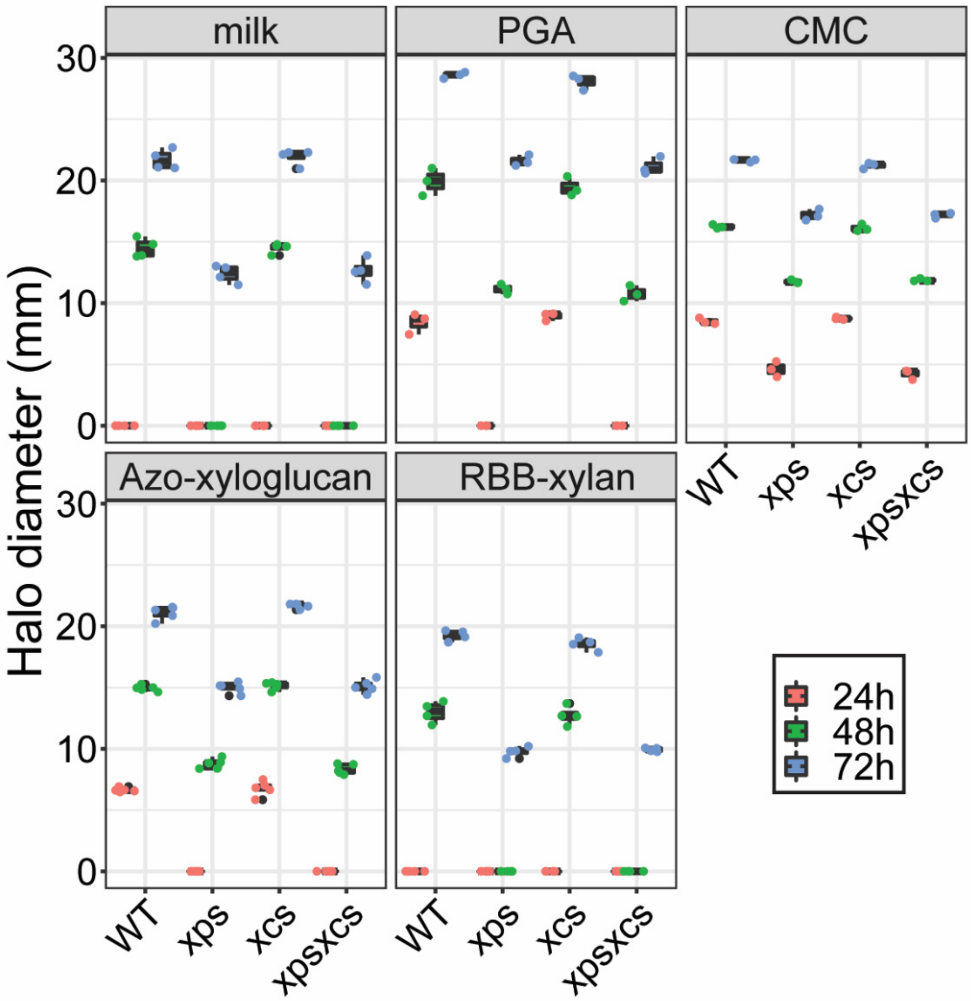

Supplementary Figure 4. Quantification of halo diameter indicating substrate degradation by *Xanthomonas*. Agar plates containing either skimmed milk, polygalacturonic acid (PGA), carboxymethyl cellulose (CMC), Azo-xyloglucan or Remazol Brilliant Blue-Xylan (RBB-Xylan). Drops of 4 µl *Xanthomonas* Leaf131 wildtype or mutant suspension were pipetted onto agar plate. Halo diameter was quantified at indicated time points. Box plots show the median with upper and lower quartiles and whiskers present 1.5× interquartile range. Representative photographs of agar plates at 24 hours are shown in Figure 3e.

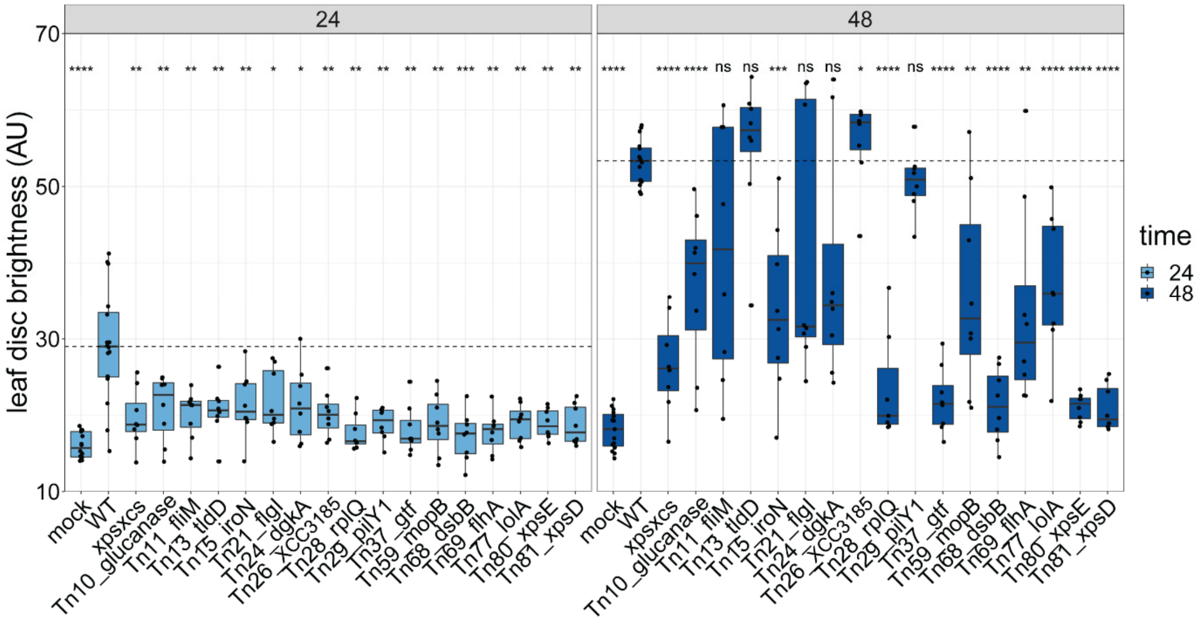

Supplementary Figure 5. Transposon mutagenesis screen revealed candidate genes involved in leaf degradation. Leaf discs of five-week-old *rbohD* plants were mock inoculated (10 mM $MgCl_2$) or with *Xanthomonas* Leaf131 wildtype (WT) or Tn mutants (OD=0.02). Box plots show the median with upper and lower quartiles and whiskers present 1.5× interquartile range. Significant difference of Tn mutants compared to WT was determined by two-sided Mann–Whitney *U*-test (n = 8) and p-values indicated as ns, non-significant; *, p<0.05, **, p<0.01; ***, p<0.001; ****, p<0.0001. Dashed line shows median of WT control.

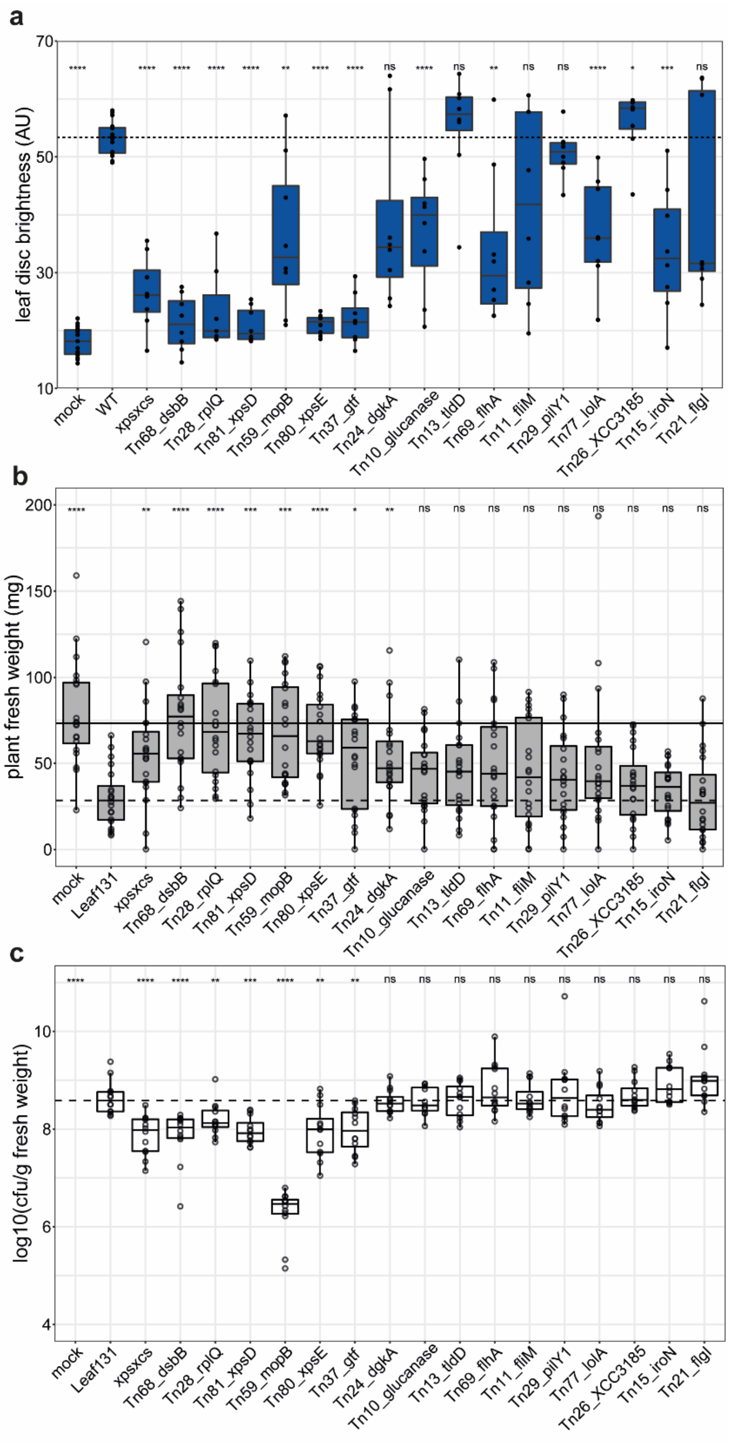

Supplementary Figure 6. Virulence of *Xanthomonas* Leaf131 transposon mutant candidates. a) Leaf discs of five-week-old *rbohD* plants were mock inoculated (10 mM $MgCl_2$) or with *Xanthomonas* Leaf131 wildtype (WT) or Tn mutants (OD=0.02) and incubated for 48 hours (same data as shown in Figure S9, 48 hours). Box plots show the median with upper and lower quartiles and whiskers present 1.5× interquartile range. Significant difference of Tn mutants compared to WT was determined by two-sided Mann–Whitney *U*-test (n = 8) and p-values indicated as ns, non-significant; *, p<0.05, **, p<0.01; ***, p<0.001; ****, p<0.0001. **b)** Fresh weight of aboveground plant tissue of five-week-old gnotobiotic *rbohD* plants either mock inoculated or with *Xanthomonas* Leaf131 wildtype or Tn mutants. **c)** Colony forming unit (CFU) counts of *Xanthomonas* Leaf131 per gram plant fresh weight from samples in b). Box plots show the median with upper and lower quartiles and whiskers present 1.5× interquartile range. Significant differences in b) (n=15) and c) (n=12) were calculated by two-sided Mann–Whitney *U*-test and p-values indicated as ns, non-significant; *, p<0.05, **, p<0.01; ***, p<0.001; ****, p<0.0001. Dashed line and solid line show median of WT control and mock control, respectively.

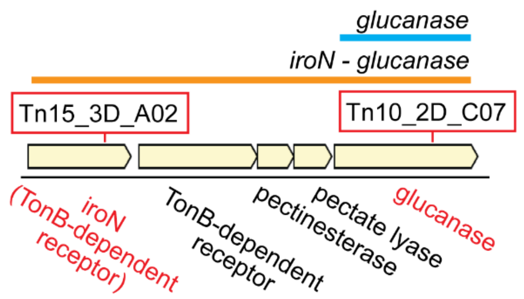

Supplementary Figure 7. Genomic region of *iroN* and *glucanase* in *Xanthomonas* Leaf131. Red boxes indicate transposon insertion in indicated Tn mutants. Orange line highlights multi-gene deletion region in strain *iroN-glucanase*. Blue line indicates single gene deletion of *glucanase*.

References

1 Kaczmarczyk, A., Hochstrasser, R., Vorholt, J. A. & Francez-Charlot, A. Complex two-component signaling regulates the general stress response in Alphaproteobacteria. *Proc. Natl. Acad. Sci.* USA **111**, E5196-E5204 (2014).

2 Das, S., Noe, J. C., Paik, S. & Kitten, T. An improved arbitrary primed PCR method for rapid characterization of transposon insertion sites. *J. Microbiol. Methods* **63**, 89-94 (2005).

Supplementary Table Legends

Supplementary Table 1. Proteomics of Leaf131 culture supernatant. a) Identification of proteins in fractions of *Xanthomonas* Leaf131 supernatant from wildtype (fraction 1 and 2) or *xpsxcs* mutant (fraction 4 and 5). **b)** Identification of proteins in fractions of *Xanthomonas* Leaf148 supernatant from wildtype (fraction 3 and 4). Fractions are protein bands excised from SDS-PAGE (Extended Data Figure 4a). Selection for gene knockout highlighted in orange. **c)** Table shows selected protein candidates for gene knockout in *Xanthomonas* Leaf131.

Supplementary Table 2. Transposon mutagenesis screen in *Xanthomonas* Leaf131. a) Overview of transposon screen and identified candidate genes. **b)** Selection of validated candidate genes.

Supplementary Table 3. SynCom strains and microbiota composition data. a) *At*-LSPHERE strains used in SynCom-137 and *Xanthomonas* Leaf131 and Leaf148. **b)** ASV count table for drop-out experiment and **c)** corresponding metadata. **d)** ASV count table for drop-in experiment and **e)** corresponding metadata.

Supplementary Table 4. Knockout strains and oligonucleotides used in this study.

Supplementary Table 5. Statistical analysis of data shown in Supplementary Figure 1. Results of two-way ANOVA of data shown in Supplementary Figure 1.
