# Supplementary material for: Leaf microbiome dysbiosis triggered by T2SS-dependent enzyme secretion from opportunistic Xanthomonas pathogens
Source: Nat Microbiol. 2024 Jan 3;9(1):136–49. doi: 10.1038/s41564-023-01555-z (PMC10769872; doi:10.1038/s41564-023-01555-z)
Supplement: Supplementary file 2 — Reporting Summary [file 41564_2023_1555_MOESM2_ESM.pdf]

nature portfolio

Corresponding author(s): Julia A Vorholt

Last updated by author(s): Oct 23, 2023

Reporting Summary

Nature Portfolio wishes to improve the reproducibility of the work that we publish. This form provides structure for consistency and transparency in reporting. For further information on Nature Portfolio policies, see our Editorial Policies and the Editorial Policy Checklist.

Statistics

For all statistical analyses, confirm that the following items are present in the figure legend, table legend, main text, or Methods section.

n/a	Confirmed	
☐	☒	The exact sample size (*n*) for each experimental group/condition, given as a discrete number and unit of measurement
☐	☒	A statement on whether measurements were taken from distinct samples or whether the same sample was measured repeatedly
☐	☒	The statistical test(s) used AND whether they are one- or two-sided<br>*Only common tests should be described solely by name; describe more complex techniques in the Methods section.*
☒	☐	A description of all covariates tested
☐	☒	A description of any assumptions or corrections, such as tests of normality and adjustment for multiple comparisons
☐	☒	A full description of the statistical parameters including central tendency (e.g. means) or other basic estimates (e.g. regression coefficient) AND variation (e.g. standard deviation) or associated estimates of uncertainty (e.g. confidence intervals)
☐	☒	For null hypothesis testing, the test statistic (e.g. *F*, *t*, *r*) with confidence intervals, effect sizes, degrees of freedom and *P* value noted<br>*Give P values as exact values whenever suitable.*
☒	☐	For Bayesian analysis, information on the choice of priors and Markov chain Monte Carlo settings
☒	☐	For hierarchical and complex designs, identification of the appropriate level for tests and full reporting of outcomes
☐	☒	Estimates of effect sizes (e.g. Cohen's *d*, Pearson's *r*), indicating how they were calculated

Our web collection on statistics for biologists contains articles on many of the points above.

Software and code

Policy information about availability of computer code

Data collection	Leaf disc brightness was measured with customized MatlabR2022a code, available at: https://github.com/gaebeleinC/leaf- 550 disc_quantification
Data analysis	Customized code for MatlabR2022a was used and deposited GitHub repository: https://github.com/gaebeleinC/leaf- 550 disc_quantification<br>Customized code to analyse data and generate figures can be found at https://github.com/MicrobiologyETHZ/phylloR<br>Data analysis was done with Rv.3.6.3. The following R packages were used during analysis and visualization: ape v.5.4, ggplot2 v.3.3.0, vegan v.2.5-4, DESeq2 v.1.14.1, ggpubr v.0.3.0

For manuscripts utilizing custom algorithms or software that are central to the research but not yet described in published literature, software must be made available to editors and reviewers. We strongly encourage code deposition in a community repository (e.g. GitHub). See the Nature Portfolio guidelines for submitting code & software for further information.

Data

Policy information about availability of data

All manuscripts must include a data availability statement. This statement should provide the following information, where applicable:

- Accession codes, unique identifiers, or web links for publicly available datasets
- A description of any restrictions on data availability
- For clinical datasets or third party data, please ensure that the statement adheres to our policy

Raw data of 16S rRNA amplicon sequencing are available at the European Nucleotide Archive: PRJEB64618.
Reference genomes of Xanthomonas Leaf131 and Xanthomonas Leaf148 accessed at the NCBI database under NCBI:txid1736270 and NCBI:txid1736275, respectively.

Research involving human participants, their data, or biological material

Policy information about studies with human participants or human data. See also policy information about sex, gender (identity/presentation), and sexual orientation and race, ethnicity and racism.

Reporting on sex and gender	not applicable
Reporting on race, ethnicity, or other socially relevant groupings	not applicable
Population characteristics	not applicable
Recruitment	not applicable
Ethics oversight	not applicable

Note that full information on the approval of the study protocol must also be provided in the manuscript.

Field-specific reporting

Please select the one below that is the best fit for your research. If you are not sure, read the appropriate sections before making your selection.

☒ Life sciences ☐ Behavioural & social sciences ☐ Ecological, evolutionary & environmental sciences

For a reference copy of the document with all sections, see nature.com/documents/nr-reporting-summary-flat.pdf

Life sciences study design

All studies must disclose on these points even when the disclosure is negative.

Sample size	Sample size for each experiment was determined based on similar experiments performed in our laboratory previously and/or described in the literature (Carlstrom et al., 2019, Nat Ecol Evol 3, 1445–1454; Pfeilmeier et al. 2021, Nat Microbiol 6, 852–864). The experimental design included a positive and/or negative control that indicate the expected effect size.
Data exclusions	No data was excluded from the analyses that satisfied criteria for quality control. For example, in 16S rRNA amplicon sequencing experiments, samples with low number of total reads were excluded. In plant colonization experiments, plants that did not survive inoculation procedure were excluded.
Replication	To assure the robustness of the biological findings, each experiment was performed independently at least twice. In addition, main results were validated by applying complementary analysis methods and by testing related phenotypes in additional experiments.
Randomization	To avoid batch effects during 16S rRNA amplicon sequencing, samples were randomized for DNA extraction and library preparation.
Blinding	The investigators were blinded to treatment group allocation during plant inoculation and plant harvest.

Reporting for specific materials, systems and methods

We require information from authors about some types of materials, experimental systems and methods used in many studies. Here, indicate whether each material, system or method listed is relevant to your study. If you are not sure if a list item applies to your research, read the appropriate section before selecting a response.

Materials & experimental systems

n/a	Involved in the study
☒	☐ Antibodies
☒	☐ Eukaryotic cell lines
☒	☐ Palaeontology and archaeology
☒	☐ Animals and other organisms
☒	☐ Clinical data
☒	☐ Dual use research of concern
☐	☒ Plants

Methods

n/a	Involved in the study
☒	☐ ChIP-seq
☒	☐ Flow cytometry
☒	☐ MRI-based neuroimaging
